# Mathematical modeling and dynamics of immunological exhaustion caused by measles transmissibility interaction with HIV host

**DOI:** 10.1371/journal.pone.0297476

**Published:** 2024-04-18

**Authors:** Dilber Uzun Ozsahin, Najeeb Alam Khan, Araib Aqeel, Hijaz Ahmad, Maged F. Alotaibi, Muhammad Ayaz

**Affiliations:** 1 Department of Medical Diagnostic Imaging, College of Health Sciences, Sharjah University, Sharjah, United Arab Emirates; 2 Research Institute for Medical and Health Sciences, University of Sharjah, Sharjah, United Arab Emirates; 3 Department of Mathematics, University of Karachi, Karachi, Pakistan; 4 Near East University, Operational Research Center in Healthcare, TRNC Mersin 10, Nicosia, Turkey; 5 Center for Applied Mathematics and Bioinformatics, Gulf University for Science and Technology, Mishref, Kuwait; 6 Department of Computer Science and Mathematics, Lebanese American University, Beirut, Lebanon; 7 Department of Physics, College of Science, King Abdulaziz University, Jeddah, Saudi Arabia; Shanxi University, CHINA

## Abstract

This paper mainly addressed the study of the transmission dynamics of infectious diseases and analysed the effect of two different types of viruses simultaneously that cause immunodeficiency in the host. The two infectious diseases that often spread in the populace are HIV and measles. The interaction between measles and HIV can cause severe illness and even fatal patient cases. The effects of the measles virus on the host with HIV infection are studied using a mathematical model and their dynamics. Analysing the dynamics of infectious diseases in communities requires the use of mathematical models. Decisions about public health policy are influenced by mathematical modeling, which sheds light on the efficacy of various control measures, immunization plans, and interventions. We build a mathematical model for disease spread through vertical and horizontal human population transmission, including six coupled nonlinear differential equations with logistic growth. The fundamental reproduction number is examined, which serves as a cutoff point for determining the degree to which a disease will persist or die. We look at the various disease equilibrium points and investigate the regional stability of the disease-free and endemic equilibrium points in the feasible region of the epidemic model. Concurrently, the global stability of the equilibrium points is investigated using the Lyapunov functional approach. Finally, the Runge-Kutta method is utilised for numerical simulation, and graphic illustrations are used to evaluate the impact of different factors on the spread of the illness. Critical factors that effect the dynamics of disease transmission and greatly affect the rate and range of the disease’s spread in the population have been determined through a thorough analysis. These factors are crucial in determining the expansion of the disease.

## 1. Introduction

A comprehensive knowledge of many elements, from microscopic host-pathogen interactions to wider ecological and social processes, is essential in our continuous fight against viral infections. The rapid spread of infectious diseases resulting from bacteria, fungi, viruses, and parasites demands a thorough investigation of their complex intrapopulation distribution. It entails determining the variables that may contribute to the disease’s spread and creating efficient treatment strategies [1–3].

Human Immunodeficiency Virus (HIV) and measles are the two main diseases we are concentrating on. These diseases are prevalent in human populations; thus, in order to effectively regulate and prevent them, a thorough investigation of their dynamics is necessary. By concentrating on the dynamics of the interaction of these two important diseases, HIV and measles, this research aims to advance our collective understanding. We seek to understand the intricacies of disease transmission with mathematical models based on ordinary differential equations.

The study of infectious disease benefits greatly from the use of mathematics, which provides crucial tools for analysis and prediction. Mathematical models provide a systematic framework for simulating the complex dynamics of disease transmission within the domain of ordinary differential equations (ODEs). Researchers may investigate several elements using this analytical technique, such as sensitivity analysis, parameter estimation, and trend predictions. The intricacy of disease dynamics is captured by mathematical models, which are crucial tools for understanding the variables affecting the progression of disease and creating successful intervention plans. Mathematics has a practical function in directing targeted therapies and shaping evidence-based public health policy, going beyond theoretical research. Essentially, using mathematical techniques improves our capacity to address the problems caused by viral diseases worldwide.

Numerous mathematical models have been created to control the development of infectious disease by studying the dynamics of the viruses [4–6]. In a particular population, epidemiology researches the prevalence and contributing factors of various health disorders. This discipline is crucial to public health because it contributes to the development of disease prevention plans and policies [7–10].

HIV has a high fatality rate with no recognised vaccine or treatment. Globally, it has infected up to 25 million individuals, and it can result in 14,000 new cases each day. The duration of AIDS is usually around 6 to 15 years. It can cause various diseases, such as cancer. The virus attacks the body’s immune system and damages CD4+ T-cells, which can lead to the development of other diseases. The main transmission route of the virus is through blood transfusions as well as sexual intercourse. HIV can be natural as well [11].

On the contrary, measles is a dangerous and potentially fatal viral infection. This pandemic has been happening for millennia in many parts of the world. According to the WHO report, 364,811 confirmed measles cases were found in 182 countries during the first quarter of 2019. Recently, in the first quarter of 2022, 2156 cases were reported globally. The measles virus is an RNA virus referred to as a paramyxovirus. It belongs to the Morbillivirus genus and the Paramyxoviridae family [12,13]. Seven to fourteen days following the initial human-host contact with the virus, the first measles symptoms that resemble the flu begin to appear. Within two to five days of the first symptoms, the person develops rashes on their body, including Koplik’s spots in their mouth, and the rash spreads to other parts of their body. Sneezing, coughing, close physical contact, exposure to contaminated nasal secretions or throat secretions, and the production of large respiratory droplets are all ways that the measles virus is transferred from one person to another [14].

HIV infection and measles co-infection result in immunodeficiency in the host. The measles virus causes a temporary immunodeficiency and cellular-mediated immunity to be suppressed [15]. Due to the live attenuated strain used in this measles vaccine, a similar but considerably milder condition of transitory immunodeficiency is predicted in the weeks after receiving the measles dose [16,17]. In contrast, a progressive condition of immunodeficiency is brought on by an HIV infection. The key component of strategies to combat or delay HIV’s effects on the immune system is the use of antiretroviral medications to prevent HIV infection [18]. People with HIV can now live virtually normal lives for lengthy periods of time due to the extensive research that has been done in this field.

Scientists can forecast the emergence and spread of infectious illnesses using mathematical models. Additionally, it can aid in deciding what social and political actions can be taken to manage the condition [19]. For instance, by calculating the number of infected individuals, the model can assist in forecasting the span of the epidemic and the different preventative actions that can be implemented. In order to comprehend the dynamics of an epidemic, it is necessary to examine a number of structural factors, including the age of the sick people, the length of the disease, and the hosts’ level of immunity [20]. Time plays an important role in this field since it gives a continuous perspective on the numerous systems that might have an impact on the epidemic [21].

In order to shed light on critical factors driving the transmission and management of the diseases, we hope to untangle the complexity surrounding HIV and measles. Our goal is to provide a better understanding of the transmission patterns, vulnerable populations, and possible preventative methods for these human-centric infections by using ordinary differential equations. We want to contribute significantly to the area of infectious disease modelling in general and to the domain of ordinary differential equations in particular, while also expanding our knowledge of HIV and measles dynamics by starting this investigation.

In this work, nonlinear differential equations are used to formalise the system. This type of model expresses the dynamics of the population by separating it into various compartments. For instance, the population of susceptible individuals is divided into six compartments. Due to the complexity of the model, various variants can be added to make it more realistic. For instance, the model considers the interaction of the measles virus with the susceptible, who are already infected with HIV.

The paper’s structure introduces the various steps involved in developing the proposed model in Section 2. The paper presents the stability analysis and epidemic thresholds of the proposed model in Section 3. Section 4 analyses the proposed model, and numerical simulations are performed to validate the results. Section 5 provides some considerations and discussion of the study.

### 2. Epidemiological modelling

**Fig 1:**
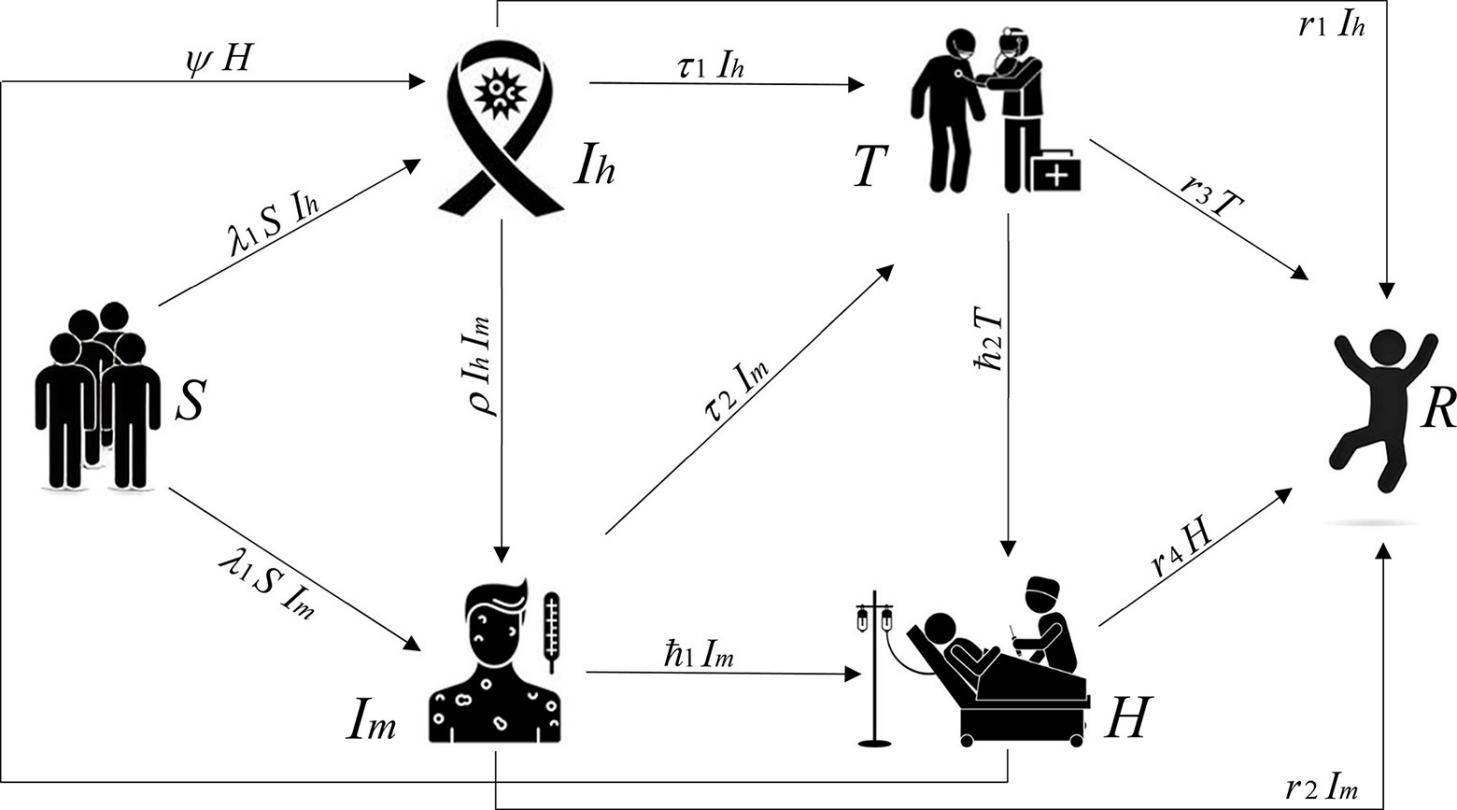
A schematic diagram of interactions between the individuals from various compartments of the *SI*_*h*_*I*_*m*_*THR* model.

We aim to describe an outbreak of the interaction between HIV and measles in a population of size N at time t. The population is divided into six groups: *S*(*t*) those who are susceptible to the diseases; *I*_*h*_(*t*) and *I*_*m*_(*t*) those who are HIV and measles infected respectively; *T*(*t*) those who are under treatment; *H*(*t*) those who are hospitalized; and finally, *R*(*t*), that represents the quantity of people who have been recovered at a given time *t*. The following postulations have been adopted in order to mathematically represent the dynamics of the system, which are also illustrated in Fig 1.

- The model assumes a uniform distribution of people in the population [22], regardless of the fact that different age groups and people with pre-existing diseases have varying risks of contracting HIV and measles.
- The infectious host of HIV and measles infects others susceptible with the rate of *λ*_1_≥0 and *λ*_2_≥0. Accordingly, the viruses are spread through interaction, the linear incidence rate of *I*_*m*_ and *I*_*h*_ is given by *λ*_1_*S I*_*m*_ and *λ*_2_*S I*_*h*_.
- The infectious host of measles co-infecting HIV-infected individuals, and the linear incidence rate is given by *ρ I*_*h*_*I*_*m*_, here *ρ*≥0 denote the co-infection rate.
- The people in compartments *I*_*m*_, *I*_*h*_, *T* and *H* are at risk of passing away from illness, which has an infection death rate of *d*_*i*_>0 (*i* = 1,2,3,4) respectively. Along with the recovery rates of *r*_*i*_>0 (*i* = 1,2,3,4) from the following compartments, they recover from illness and develop immunity.
- The infected individuals in *I*_*h*_ and *I*_*m*_ compartments are going under treatment to recover from the diseases at the rate of *τ*_1_>0 and *τ*_2_>0.
- The co-existence of the diseases HIV and measles causes severely sick and fatal cases that are existing in *I*_*m*_ and *T* compartments, being hospitalized at the rates of ℏ_1_>0 and ℏ_2_>0 severally.
- The hospitalized people who are co-infected, develop immunity and recover from the measles disease but are still infected with HIV are moving back to the *I*_*h*_ compartment with rate of *ψ*>0 as the infection phase of measles normally lasts 8–12 days, but HIV takes a long period of time to recover.
- Individuals in the host population experience natural mortality at a certain rate *d*_*i*_>0 (*i* = 0.5).

These propositions allow the dynamics of the aforementioned real-world system to be mathematically represented as:

$$N(t)=S(t)+{Ι}_{h}(t)+{Ι}_{m}(t)+T(t)+H(t)+R(t),$$ (1)

$$\begin{array}{l} \frac{dS}{dt}=rS(1-\frac{S}{k})-\lambda_{1}S\;I_{m}-\lambda_{2}S\;I_{h}-d_{0}S,\\ \frac{dI_{h}}{dt}=\lambda_{2}S\;I_{h}-\rho I_{h}I_{m}-(\tau_{1}+r_{1}+d_{1})I_{h}+\psi H,\\ \frac{dI_{m}}{dt}=\lambda_{1}SI_{m}+\rho I_{h}I_{m}-(\tau_{2}+\hbar_{1}+r_{2}+d_{2})I_{m},\\ \frac{dT}{dt}=\tau_{1}I_{h}+\tau_{2}I_{m}-(\hbar_{2}+r_{3}+d_{3})T,\\ \frac{dH}{dt}=\hbar_{1}I_{m}+\hbar_{2}T-(r_{4}+d_{4}+\psi )H,\\ \frac{dR}{dt}=r_{1}I_{h}+r_{2}I_{m}+r_{3}T+r_{4}H-d_{5}R, \end{array}$$ (2)

with initial condition,

$$S(0)\geq 0,I_{h}(0)\geq 0,I_{m}(0)\geq 0,T(0)\geq 0,H(0)\geq 0,R(0)\geq 0.$$

**Theorem 1**. *Assume $\delta \in R_{+}^{6}$ is the set of the system (2) that contains all the feasible solutions, then there exists positively invariant and uniformly bounded subset of $R_{+}^{6}$ such that:*

$$\delta =\{(S,I_{h},I_{m},T,H,R)\in R_{+}^{6}:N(t)\leq \frac{r}{d_{n}k}\},$$

To prove [23], taking time derivative of Eq (1), obtaining the expression:

$$\frac{dN(t)}{dt}=\frac{d}{dt}(S(t)+I_{h}(t)+I_{m}(t)+T(t)+H(t)+R(t)).$$ (3)

In order to simplify Eq 3 by using system (2), let assume *d*_*n*_ is the overall rate of deaths across all compartments [24] i.e.


$$\frac{dN(t)}{dt}\leq rS(1-\frac{S}{k})-d_{n}N,$$ (4)

Where $0<\frac{S}{k}\leq 1,$ so the Eq (4) reduce to

$$\frac{dN(t)}{dt}\leq \frac{r}{k}-d_{n}N,$$ (5)

When integrating

$$N(t)\leq \frac{r}{d_{n}k}+e^{-dnt}c,$$ (6)

$$N(t)\leq \frac{r}{d_{n}k}+e^{-dnt}(N(0)-\frac{r}{d_{n}k}).$$ (7)

And *e*^−*dnt*^→0 as *t*→∞, so $\lim_{t\to \infty }N(t)\leq \frac{r}{d_{n}k}.$ Hence, the feasible region of the system (2) is

$$\delta =\{(S,I_{h},I_{m},T,H,R)\in R_{+}^{6}:N(t)\leq \frac{r}{d_{n}k}\}.$$ (8)

Since all solutions either stay in or tend to the feasible area *δ*, it is easy to recognize that this is the positive invariant set for the system (2).

## 3. Stability analysis and epidemic thresholds

One of the most important factors in analysing the solutions in a system is the equilibrium that doesn’t change over time. For instance, in compartmental models, two such solutions are required: the Free Disease Equilibrium and the Endemic Equilibrium [21].This section primarily focuses on presenting the preliminary findings and computing the reproduction number *R*_0_, a crucial threshold quantity for predicting the sustainability of diseases in a population. The first equilibrium point is the disease-free equilibrium (DFE) point, that is

$$E^{0}(S^{0},{I_{h}}^{0},{I_{m}}^{0},T^{0},H^{0})=(\frac{k(r-d_{0})}{r},0,0,0,0,0).$$ (9)

The largest eigenvalue of the following next generation matrix (12) is the fundamental reproduction number of model (2) [25], represented by (i.e., the largest of the reproduction numbers, as determined by each disease). It can be acquired using the technique below.


$$F=[\begin{array}{cccc} S\;\lambda_{2}-I_{m}\rho & 0 & 0 & 0\\ 0 & S\;\lambda_{1}+I_{h}\rho & 0 & 0\\ 0 & 0 & 0 & 0\\ 0 & 0 & 0 & 0 \end{array}],$$ (10)

$$U=[\begin{array}{cccc} \tau_{1}+r_{1}+d_{1} & 0 & 0 & 0\\ 0 & \tau_{2}+r_{2}+\hbar_{1}+d_{2} & 0 & 0\\ -\tau_{1} & -\tau_{2} & r_{3}+\hbar_{2}+d_{3} & 0\\ 0 & -\hbar_{1} & r_{4}+d_{4} & 0 \end{array}],$$ (11)

We find that

$$FU^{-1}(E^{0})=[\begin{array}{cccc} \frac{(r-d_{0})k\lambda_{2}}{r(\tau_{1}+r_{1}+d_{1})} & 0 & 0 & 0\\ 0 & \frac{(r-d_{0})k\lambda_{1}}{r(\tau_{2}+r_{2}+d_{2}+\hbar_{1})} & 0 & 0\\ 0 & 0 & 0 & 0\\ 0 & 0 & 0 & 0 \end{array}].$$ (12)

The following threshold quantity of the model can be determined by the spectral radius of matrix *FU*^−1^(*E*^0^),

$$R1=\frac{(r-d_{0})k\lambda_{1}}{r(\tau_{2}+r_{2}+\hbar_{1}+d_{2})}\;\mathrm{and}\;R_{2}=\frac{(r-d_{0})k\lambda_{2}}{r(\tau_{1}+r_{1}+d_{1})}.$$ (13)

$$R_{0}=\max\{R_{1},R_{2}\}.$$ (14)

Furthermore, the DFE and the EE are utilized in order to study their global and local stability. For the DFE, the linearized system’s characteristic equation has roots with a negative real part. This explains the local stability of the FDE. Local stability of the equilibrium point can be determined using the Routh-Hurwitz criterion [26].

However, The global stability of the DFE and the EE can be determined through the Lyapunov function; Several authors have already developed a general form of the Lyapunov function that can be used in various epidemiological models which can be found under the same assumptions as the *R*_0_ problem [27]. This method is utilized to prove the global stability of the systems (2).

**Theorem 2.** *The system (2) disease-free equilibrium point E*^0^(*S*^0^,0,0,0,0) *is LAS when R*_0_<1 *and unstable for other conditions*.


$$J(E^{0})=[\begin{array}{cccccc} d_{0}-r & \frac{(d_{0}-r)k\lambda_{2}}{r} & \frac{(d_{0}-r)k\lambda_{1}}{r} & 0 & 0 & 0\\ 0 & -d_{1}-r_{1}-\frac{(d_{0}-r)k\lambda_{2}}{r}-\tau_{1} & 0 & 0 & \psi & 0\\ 0 & 0 & -d_{2}-r_{2}-\frac{(d_{0}-r)k\lambda_{1}}{r}-\tau_{2}-\hbar_{1} & 0 & 0 & 0\\ 0 & \tau_{1} & \tau_{2} & -d_{3}-r_{3}-\hbar_{2} & 0 & 0\\ 0 & 0 & \hbar_{1} & \hbar_{2} & -d_{4}-\psi -r_{4} & 0\\ 0 & r_{1} & r_{2} & r_{3} & r_{4} & -d_{5} \end{array}]$$ (15)

The characteristic polynomial equation of *J*(*E*^0^) is

$$\begin{array}{l} \left(\omega +d_{5})(-d0+\omega +r)(d_{2}+\omega +r_{2}+\frac{k(r-d_{0})\lambda_{1}}{r}+\tau_{2}+\hbar_{1}\right)\\ (-\psi r\tau_{1}\hbar_{2}+(d_{4}+\psi +\omega +r_{4})(rd_{1}+r\omega +rr_{1}+d_{0}k\lambda_{2}-rk\lambda_{2}+r\tau_{1})(d_{3}+\omega +r_{3}+\hbar_{2}))=0. \end{array}$$ (16)

The eigenvalues of the Jacobian are *ω*_1_ = −*d*_5_, *ω*_2_ = *d*_0_−*r*, $\omega_{3}=-(d_{2}+r_{2}+\tau_{2}+\hbar_{2})(1-R_{1})$ and the roots of the characteristic polynomial arising from the remaining minor of order three:

$$\Delta (\omega )=\omega^{3}+\omega^{2}m_{2}+\omega m_{1}+m_{0}=0,$$ (17)

$$\begin{array}{l} m_{0}=(1-R_{2})(d_{1}+r_{1}+\tau_{1})(d_{3}d_{4}+d_{3}\psi +d_{4}r_{3}+\psi r_{3}+d_{3}r_{4}+r_{3}r_{4}+d_{4}\hbar_{2}+\psi \hbar_{2}+r_{4}\hbar_{2})\\ \;-\psi \tau_{1}\hbar_{2}, \end{array}$$ (18)

$$m_{1}=(1-R_{2})(d_{1}+r_{1}+\tau_{1})+d_{3}+\psi +d_{4}+r_{3}+r_{4}+\hbar_{2},$$ (19)

$$\begin{array}{l} m_{2}=(1-R_{2})(d_{1}+r_{1}+\tau_{1})(d_{3}+\psi +d_{4}+r_{3}+r_{4}+\hbar_{2})\\ \;+(d_{3}d_{4}+d_{3}\psi +d_{4}r_{3}+\psi r_{3}+d_{3}r_{4}+r_{3}r_{4}+d_{4}\hbar_{2}+\psi \hbar_{2}+r_{4}\hbar_{2}), \end{array}$$ (20)

Since, $E^{0}\in R_{+}^{6}$ is asymptotically stable if *R*_0_<1. Also

$$\begin{array}{l} \left(1-R_{2})(d_{1}+r_{1}+\tau_{1})(d_{3}d_{4}+d_{3}\psi +d_{4}r_{3}+\psi r_{3}+d_{3}r_{4}+r_{3}r_{4}+d_{4}\hbar_{2}+\psi \hbar_{2}+r_{4}\hbar_{2}\right)\\ \;>\psi \tau_{1}\hbar_{2}. \end{array}$$

Regarding cubic polynomial Routh-Hurwitz criteria [28], if *m*_0_>0,*m*_2_>0 and *m*_1_ *m*_2_<*m*_3_, then Δ(*ω*)>0 and thus the real part of each eigenvalue must all be negative of Eq (17).It is obvious to observe that *m*_0_>0,*m*_1_>0 and *m*_2_>0. Hence $E^{0}\in R_{+}^{6}$ is asymptotically stable. And if *R*_0_>1 then (1−*R*_0_)<0 implies Δ(*ω*)<0 that is Eq (17) must have a nonnegative real part, thus $E^{0}\in R_{+}^{6}$ becomes unstable.

**Theorem 3.** *The system (2)Trivial point $E^{T}(0,0,0,0,0)$ is locally asymptotically unstable.*


$$J(E^{T})=[\begin{array}{cccccc} r-d_{0} & 0 & 0 & 0 & 0 & 0\\ 0 & -d_{1}-r_{1}-\tau_{1} & 0 & 0 & 0 & 0\\ 0 & 0 & -d_{2}-r_{2}-\tau_{2}-\hbar_{1} & 0 & 0 & 0\\ 0 & \tau_{1} & \tau_{2} & -d_{3}-r_{3}-\hbar_{2} & 0 & 0\\ 0 & 0 & \hbar_{1} & \hbar_{2} & -d_{4}-r_{4} & 0\\ 0 & r_{1} & r_{2} & r_{3} & r_{4} & -d_{5} \end{array}],$$ (21)

The characteristic polynomial equation of *J*(*E*^*T*^) is

$$\begin{array}{l} \left(\omega +d_{5})(d_{0}+\omega -r)(d_{2}+\omega +r_{2}+\tau_{2}+\hbar_{1}\right)\\ (-\psi \tau_{1}\hbar_{2}+(d_{4}+\psi +\omega +r_{4})(d_{1}+\omega +r_{1}+\tau_{1})(d_{3}+\omega +r_{3}+\hbar_{2}))=0. \end{array}$$ (22)

The simplified eigenvalues are *ω*_1_ = −*d*_5_, *ω*_2_ = *r*−*d*_0_, *ω*_3_ = −*d*_2_−*r*_2_−*τ*_2_−ℏ_1_ and the other three roots will be determine by the equation

$$(-\psi \tau_{1}\hbar_{2}+(d_{4}+\psi +\omega +r_{4})(d_{1}+\omega +r_{1}+\tau_{1})(d_{3}+\omega +r_{3}+\hbar_{2}))=0.$$ (23)

It is shown that the second eigenvalue of Jacobian is $\omega_{2}=r-d_{0}$ at *E*^*T*^ is nonnegative. Since *r*>*d*_0_, so it is clear *ω*_2_>0. Thus $E^{T}\in R_{+}^{6}$ is unstable.

**Theorem 4** *The Endemic point equilibrium $E^{*}(S^{*},{I_{h}}^{*},{I_{m}}^{*},T^{*},H^{*})$ is LAS when R*_0_>1 *and otherwise unstable*, *and the eigenvalues ω*_*i*_ *of the Jacobian matrix J*(E*) *that satisfy the aforementioned requirements in [29].*


$$J(E*)=[\begin{array}{cccccc} -d_{0}+r-\frac{2rS^{*}}{k}-\lambda_{1}{I_{m}}^{*}-\lambda_{2}{I_{h}}^{*} & -S^{*}\lambda_{2} & -S^{*}\lambda_{1} & 0 & 0 & 0\\ \lambda_{2}{I_{h}}^{*} & -d_{1}-r_{1}+S^{*}\lambda_{2}-\rho {I_{m}}^{*}-\tau_{1} & -\rho {I_{h}}^{*} & 0 & \psi & 0\\ \lambda_{1}{I_{m}}^{*} & \rho {I_{m}}^{*} & -d_{2}-r_{2}+S^{*}\lambda_{1}+\rho {I_{h}}^{*}-\tau_{2}-\hbar_{1} & 0 & 0 & 0\\ 0 & \tau_{1} & \tau_{2} & -d_{3}-r_{3}-\hbar_{2} & 0 & 0\\ 0 & 0 & \hbar_{1} & \hbar_{2} & -d_{4}-\psi -r_{4} & 0\\ 0 & r_{1} & r_{2} & r_{3} & r_{4} & -d_{5} \end{array}],$$ (24)

The characteristic polynomial equation of *J*(E*) is

$$(\omega +d_{5})(\begin{array}{l} (C_{1+\omega })(C_{2+\omega })\left(\begin{array}{l} \left(d_{2}+A_{1})A_{3}(L_{1}-\rho A_{2}+k{\lambda_{1}}^{2}\omega )+B_{3}+P\rho A_{3}k\lambda_{1}(A_{1}\lambda_{2}+\rho \omega \right)\\ -\omega (B_{2}-(d_{2}r+k\lambda_{1}\omega +rA_{1})(-L_{1}+k\lambda_{1}d_{0}\rho -k{\lambda_{1}}^{2}\omega )) \end{array}\right)+\\ \psi \left(\begin{array}{l} (-\tau_{2}A_{3}(B_{1}-k\lambda_{1}(A_{1}\lambda_{2}-\rho \omega ))-k{\lambda_{1}}^{2}\tau_{1}(-\omega (d_{2}r+k\lambda_{1}\omega +rA_{1})+(d_{2}+A_{1})A_{3}))\hbar_{2}\\ -\hbar_{1}A_{3}(B_{1}-k\lambda_{1}(A_{1}\lambda_{2}-\rho \omega ))(C_{2}+\omega ) \end{array}\right) \end{array})=0,$$ (25)

The eigenvalues of the Jacobian are *ω*_1_ = −*d*_5_ and the roots of the characteristic polynomial arising from the remaining minor of order three:

$$ℜ(\omega )=\omega^{5}+\omega^{4}n_{4}+\omega^{3}n_{3}+\omega^{2}n_{2}+\omega n_{1}+n_{0}=0.$$ (26)

$$n_{0}=\frac{1}{k^{2}{\lambda_{1}}^{3}}(\begin{array}{l} -C_{1}C_{2}(B_{3}+(A_{1}A_{2}kP\lambda_{1}\lambda_{2}\rho +C_{3}(L_{1}-A_{2}\rho )))\\ +\psi (C_{3}k{\lambda_{1}}^{2}\tau_{1}\hbar_{2}+A_{3}(B_{1}-A_{1}k\lambda_{1}\lambda_{2})(C_{2}\hbar_{1}+\tau_{2}\hbar_{2})) \end{array}),$$ (27)

$$n_{1}=-\frac{1}{k^{2}{\lambda_{1}}^{3}}(\begin{array}{l} \left(B_{3}+C_{3}L1-A_{2}C_{3}\rho +A_{1}A_{3}kP\lambda_{1}\lambda_{2}\rho )(C_{1}+C_{2}\right)\\ +C_{1}C_{2}(-B_{2}-A_{1}L_{1}r-d_{2}L_{1}r+C_{3}k{\lambda_{1}}^{2}+d_{0}kr\lambda_{1}\rho (A_{1}+d_{2})+A_{3}kP\lambda_{1}\rho^{2})\\ -A_{3}\hbar_{1}\psi (B_{1}+k\lambda_{1}(A_{1}\lambda_{2}-C_{2}\rho ))+\psi k\lambda_{1}((A_{1}+d_{2})r\lambda_{1}\tau_{1}-A_{3}\rho \tau_{2})\hbar_{2} \end{array}),$$ (28)

$$n_{2}=-\frac{1}{k^{2}{\lambda_{1}}^{3}}(\begin{array}{l} B_{3}+(C_{1}+C_{2})(-B_{2}-L_{1}r(A_{1}+d_{2})+C_{3}k{\lambda_{1}}^{2}+d_{2}d_{0}kr\lambda_{1}\rho +A_{3}kP\lambda_{1}\rho^{2})\\ +C_{3}(L_{1}-A_{2}\rho )+C_{1}C_{2}k\lambda_{1}(-L_{1}-r\lambda_{1}(A_{1}+d_{2})+d_{0}k\lambda_{1}\rho )\\ +A_{1}k\lambda_{1}((C_{1}+C_{2})d_{0}r+A_{3}P\lambda_{2})\rho +\psi k\lambda_{1}(-A_{3}\rho \hbar_{1}-k{\lambda_{1}}^{2}\tau_{1}\hbar_{2}) \end{array}),$$ (29)

$$n_{3}=\frac{1}{k^{2}{\lambda_{1}}^{3}}(\begin{array}{l} B_{2}+k\lambda_{1}((C_{1}+C_{2})(-L_{1}+d_{0}k\lambda_{1}\rho )-C_{3}\lambda_{1}+C_{1}C_{2}k{\lambda_{1}}^{2}-A_{3}P\rho^{2})\\ +(L_{1}+k\lambda_{1}((C_{1}+C_{2})\lambda_{1}-d_{0}\rho ))(A_{1}+d_{2})r \end{array}),$$ (30)

$$n_{4}=\frac{1}{k{\lambda_{1}}^{2}}(L_{1}+\lambda_{1}((A_{1}+d_{2})r+(C_{1}+C_{2})k\lambda_{1}-d_{0}k\rho )),$$ (31)

Regarding order five polynomial Routh-Hurwitz criteria by Hurwitz matrix [30],if *n*_*i*_>0(*i* = 0,1,2,3,4) and

$n_{0}n_{1}n_{2}n_{3}n_{4}+{n_{0}}^{2}n_{2}n_{3}+2{n_{0}}^{2}n_{1}n_{4}>{n_{0}}^{2}{n_{3}}^{2}n_{4}+n_{0}{n_{1}}^{2}{n_{4}}^{2}+n_{0}n_{1}{n_{2}}^{2}+{n_{0}}^{3}$ if and only if *R*_0_>1 then ℜ(*ω*)>0, implies that the real part of each eigenvalue must all be negative of Eq (26). Hence $E^{*}\in R_{+}^{6}$ is asymptotically stable if *R*_0_>1.

**Theorem 5.** *((Existence and Uniqueness))Consider the real-valued function as the matrix of the right hand side of system (2) Ω(t,W(t)): $R_{+}^{6}\to R_{+}^{6}$, such that Ω(t,W(t)) and $\frac{\partial \Omega (t,W(t))}{\partial W(t)}$ are continuous and ‖Ω(t,W(t))‖≤N‖W(t)‖, $\forall \Omega (t,W(t))\in R_{+}^{6}$ and N are constants with positive value.*

First, we show that the suggested model (2) provided here has a singular solution for all initial circumstances in $R_{+}^{6}$. The aforementioned conditions above are categorically satisfied by the vector function Ω of the model (2). For the last condition we rearranging the proposed model (2) in the following manner

$$\begin{array}{r} \frac{dW(t)}{dt}=M_{1}W(t)+M_{2}S(t)W(t)+M_{3}I_{h}(t)W(t)+M_{4}I_{m}(t)W(t)+M_{5}T(t)W(t)\\ +M_{6}H(t)W(t)+M_{7}R(t)W(t), \end{array}$$ (32)

the matrices are provided by

$$M_{1}=[\begin{array}{cccccc} r-d_{0} & 0 & 0 & 0 & 0 & 0\\ 0 & -d_{1}-r_{1}-\tau_{1} & 0 & 0 & 0 & 0\\ 0 & 0 & -d_{2}-r_{2}-\tau_{2}-\hbar_{1} & 0 & 0 & 0\\ 0 & \tau_{1} & \tau_{2} & -d_{3}-r_{3}-\hbar_{2} & 0 & 0\\ 0 & 0 & \hbar_{1} & \hbar_{2} & -d_{4}-r_{4} & 0\\ 0 & r_{1} & r_{2} & r_{3} & r_{4} & -d_{5} \end{array}],$$ (33)

$M_{2}=[\begin{array}{cccccc} \frac{-r}{k} & 0 & 0 & 0 & 0 & 0\\ 0 & \lambda_{2} & 0 & 0 & 0 & 0\\ 0 & 0 & \lambda_{1} & 0 & 0 & 0\\ 0 & 0 & 0 & 0 & 0 & 0\\ 0 & 0 & 0 & 0 & 0 & 0\\ 0 & 0 & 0 & 0 & 0 & 0 \end{array}]$, $M_{3}=[\begin{array}{cccccc} -\lambda_{2} & 0 & 0 & 0 & 0 & 0\\ 0 & 0 & 0 & 0 & 0 & 0\\ 0 & 0 & \rho & 0 & 0 & 0\\ 0 & 0 & 0 & 0 & 0 & 0\\ 0 & 0 & 0 & 0 & 0 & 0\\ 0 & 0 & 0 & 0 & 0 & 0 \end{array}]$, $M_{4}=[\begin{array}{cccccc} -\lambda_{1} & 0 & 0 & 0 & 0 & 0\\ 0 & 0 & 0 & 0 & 0 & 0\\ 0 & 0 & -\rho & 0 & 0 & 0\\ 0 & 0 & 0 & 0 & 0 & 0\\ 0 & 0 & 0 & 0 & 0 & 0\\ 0 & 0 & 0 & 0 & 0 & 0 \end{array}]$,

*M*_5_ = [0]_6×6_, *M*_5_ = [0]_6×6_, *M*_5_ = [0]_6×6_.

This can be expressed further as follows

$$\begin{array}{l} \Omega (t,W(t))=M_{1}W(t)+M_{2}S(t)W(t)+M_{3}I_{h}(t)W(t)+M_{4}I_{m}(t)W(t)+M_{5}T(t)W(t)\\ \;+M_{6}H(t)W(t)+M_{7}R(t)W(t). \end{array}$$ (34)

Adding the norm, we have

$$\begin{array}{l} \left\|\Omega (t,W(t))\|=\|M_{1})W(t)+M_{2}S(t)W(t)+M_{3}I_{h}(t)W(t)+M_{4}I_{m}(t)W(t)+M_{5}T(t)W(t\right)\\ \;+M_{6}H(t)W(t)+M_{7}R(t)\|W(t)\|, \end{array}$$ (35)

$$\begin{array}{l} \left\|\Omega (t,W(t))\|\leq \|M_{1}\|\|W(t)\|+\|M_{2}\|\|S(t)\|\|W(t)\|+\|M_{3}\|\|I_{h}(t)\|\|W(t)\|+\|M_{4}\|\|I_{m}(t)\|\|W(t)\right\|\\ \;+\|M_{5}\|\|T(t)\|\|W(t)\|+\|M_{6}\|\|H(t)\|\|W(t)\|+\|M_{7}\|\|R(t)\|\|W(t)\|, \end{array}$$ (36)

$$\begin{array}{l} \left\|\Omega (t,W(t))\|\leq \|M_{1}\|\|W(t)\|+\|M_{2}\|\|W(t)\|+\|M_{3}\|\|W(t)\|+\|M_{4}\|\|W(t)\|+\|M_{5}\|\|W(t)\right\|\\ \;+\|M_{6}\|\|W(t)\|+\|M_{7}\|\|W(t)\|, \end{array}$$ (37)

$$\|\Omega (t,W(t))\|\leq (\left\|M_{1}\|+\|M_{2}\|+\|M_{3}\|+\|M_{4}\|+\|M_{5}\|+\|M_{6}\|+\|M_{7}\right\|)\|W(t)\|,$$ (38)

‖Ω(t,W(t))‖≤N‖W(t)‖. (39)

As a result, the last condition of the theorem is satisfied, and the suggested model (2) has a unique solution.

Now, the positivity of the initial conditions of system (2) is used to demonstrate the non-negativity of the solutions. It can be inferred from the first equation of the system (2) to

$$\frac{dS(t)}{dt}=rS(t)(1-\frac{S(t)}{k})-\lambda_{1}S(t)I_{m}(t)-\lambda_{2}S(t)I_{h}(t)-d_{0}S(t),$$

$$\geq -d_{0}S(t).$$

On manipulating, we obtain

$$S(t)\geq \varsigma_{1}e^{-d_{0}t},$$ (40)

Since $0\leq \varsigma_{1}e^{-d_{0}t}\leq 1$for *t*>0,

Therefore, *S*(*t*)≥0.

The non-negativity of *S*(*t*) has been demonstrated. Similar to this, it is possible to demonstrate that all of the remaining equations in system (2) have non-negative solutions under the premise of positive initial conditions.

**Theorem 6.** *Assume that DFE E*^0^(*S*^0^,0,0,0,0) *of model (2) is LAS*, *then it is globally asymptotically stable in Ω*.

To prove, consider a Lyapunov function

$$L_{f}=S-S^{0}-S^{0}\ln\frac{S}{S^{0}}+I_{h}+I_{m}+T+H.$$ (41)

Using the solution of system (2) to compute the derivative of Eq (41) with respect to time.


$$\frac{dL_{f}}{dt}=(\frac{S-S^{0}}{S})\frac{dS}{dt}+\frac{d}{dt}(I_{h}+I_{m}+T+H),$$ (42)

Then, this might be rewritten as

$$\begin{array}{l} \frac{dL_{f}}{dt}=(\frac{S-S^{0}}{S})(rS(1-\frac{S}{k})-\lambda_{1}S\;I_{m}-\lambda_{2}SI_{h}-d_{0}S)+I_{h}(\lambda_{2}S-r_{1}-d_{1})\\ \;+I_{m}(\lambda_{1}S-r_{2}-d_{2})-T(r_{3}+d_{3})-H(r_{4}+d_{4}), \end{array}$$ (43)

Following some simplification, we now have

$$\begin{array}{l} =(\frac{S-S^{0}}{S})(\frac{S}{S^{0}}(d_{0}-r)(S-S^{0}))+(\lambda_{2}S^{0}-r_{1}-d_{1})I_{h}\\ \;+(\lambda_{1}S^{0}-r_{2}-d_{2})I_{m}-(r_{3}+d_{3})T-(r_{4}+d_{4})H, \end{array}$$ (44)

$$\begin{array}{l} =-\frac{(r-d_{0}){\left(S-S^{0}\right)}^{2}}{S^{0}}+(\frac{\lambda_{2}S^{0}}{r_{1}+d_{1}}-1)(r_{1}+d_{1})I_{h}+(\frac{\lambda_{1}S^{0}}{r_{2}+d_{2}}-1)(r_{2}+d_{2})I_{m}\\ \;-(r_{3}+d_{3})T-(r_{4}+d_{4}). \end{array}$$ (45)

If $\frac{\lambda_{2}S^{0}}{r_{1}+d_{1}}<1$ and $\frac{\lambda_{2}S^{0}}{r_{1}+d_{2}}<1$ then *R*_0_<1, We get $\frac{dL_{f}}{dt}<0$. Thus $\frac{dL_{f}}{dt}=0$ if and only if *S* = *S*^0^, *I*_*h*_ = *I*_*m*_ = *T* = *H* = 0. Therefore, *E*^0^ is the greatest compact invariant set for system (2). The Lyapunov LaSalle stability theorem follows. We conclude that in the positive region $R_{+}^{6}\geq 0$, *E*^0^ is globally asymptotically stable for *R*_0_<1.

**Theorem 7.** *The system (2) endemic equilibrium $E^{*}(S^{*},{I_{h}}^{*},{I_{m}}^{*},T^{*},H^{*})$ is globally asymptomatically stable when R*_0_ *is larger than unity*.

To prove, consider a Lyapunov function

$$\begin{array}{l} L_{f}=(S-S^{*}-S^{*}\ln\frac{S}{S^{*}})+(I_{h}-{I_{h}}^{*}-{I_{h}}^{*}\ln\frac{I_{h}}{{I_{h}}^{*}})+(I_{m}-{I_{m}}^{*}-{I_{m}}^{*}\ln\frac{I_{m}}{{I_{m}}^{*}})\\ \;+(T-T^{*}-T^{*}\ln\frac{T}{T^{*}})+(H-H^{*}-H^{*}\ln\frac{H}{H^{*}}). \end{array}$$ (46)

Taking *L*_*f*_ time derivative results in

$$\begin{array}{l} \frac{dL_{f}}{dt}=(\frac{S-S^{*}}{S})\frac{dS}{dt}+(\frac{I_{h}-{I_{h}}^{*}}{I_{h}})\frac{dI_{h}}{dt}+(\frac{I_{m}-{I_{m}}^{*}}{I_{m}})\frac{dI_{m}}{dt}\\ +(\frac{T-T^{*}}{T})\frac{dT}{dt}+(\frac{H-H^{*}}{H})\frac{dH}{dt}, \end{array}$$ (47)

Then, this might be rewritten as

$$\begin{array}{l} \frac{dL_{f}}{dt}=(\frac{S-S^{*}}{S})(rS(1-\frac{S}{k})-\lambda_{1}SI_{m}-\lambda_{2}SI_{h}-d_{0}S)\\ \;+(\frac{I_{h}-{I_{h}}^{*}}{I_{h}})(\lambda_{2}SI_{h}-\rho I_{h}I_{m}-(\tau_{1}+r_{1}+d_{1})I_{h}+\psi H)\\ \;+(\frac{I_{m}-{I_{m}}^{*}}{I_{m}})(\lambda_{1}SI_{m}+\rho I_{h}I_{m}-(\tau_{2}+\hbar_{1}+r_{2}+d_{2})I_{m})\\ \;+(\frac{T-T^{*}}{T})(\tau_{1}I_{h}+\tau_{2}I_{m}-(\hbar_{2}+r_{3}+d_{3})T)\\ \;+(\frac{H-H^{*}}{H})(\hbar_{1}I_{m}+\hbar_{2}T-(r_{4}+d_{4}+\psi )H), \end{array}$$ (48)

Following some simplification, we now have

$$\begin{array}{l} \frac{dL_{f}}{dt}=(\frac{S-S^{*}}{S^{*}})(-(S-S^{*})(r-d_{0})+S({I_{m}}^{*}\lambda_{1}+{I_{h}}^{*}\lambda_{2})-S^{*}(I_{m}\lambda_{1}+I_{h}\lambda_{2}))\\ \;+(\frac{I_{h}-{I_{h}}^{*}}{I_{h}})\left(\psi (\frac{H{I_{h}}^{*}-H^{*}I_{h}}{{I_{h}}^{*}})+I_{h}\lambda_{2}(S-S^{*})-I_{h}\rho (I_{m}-{I_{m}}^{*})\right)\\ \;+(I_{m}-{I_{m}}^{*})(\lambda_{1}(S-S^{*})+\rho (I_{h}-{I_{h}}^{*}))\\ \;+(\frac{T-T^{*}}{T})(\tau_{1}I_{h}+\tau_{2}I_{m})-(\frac{T-T^{*}}{T^{*}})(\tau_{1}{I_{h}}^{*}+\tau_{2}{I_{m}}^{*})\\ \;+(\frac{H-H^{*}}{H})(\hbar_{1}I_{m}+\hbar_{2}T)-(\frac{H-H^{*}}{H^{*}})(\hbar_{1}{I_{m}}^{*}+\hbar_{2}T^{*}), \end{array}$$ (49)

Further simplifying

$$\begin{array}{l} \frac{dL_{f}}{dt}=-\frac{{\left(S-S^{*}\right)}^{2}}{S^{*}}(r-d_{0})+\frac{{\left(S-S^{*}\right)}^{2}}{S^{*}}({I_{m}}^{*}\lambda_{1}+{I_{h}}^{*}\lambda_{2})\\ \;+\psi H(\frac{I_{h}-{I_{h}}^{*}}{I_{h}})-\psi H^{*}(\frac{I_{h}-{I_{h}}^{*}}{{I_{h}}^{*}})\\ \;+(\frac{T-T^{*}}{T})(\tau_{1}I_{h}+\tau_{2}I_{m})-(\frac{T-T^{*}}{T^{*}})(\tau_{1}{I_{h}}^{*}+\tau_{2}{I_{m}}^{*})\\ \;+(\frac{H-H^{*}}{H})(\hbar_{1}I_{m}+\hbar_{2}T)-(\frac{H-H^{*}}{H^{*}})(\hbar_{1}{I_{m}}^{*}+\hbar_{2}T^{*}). \end{array}$$ (50)

Therefore $\frac{dL_{f}}{dt}=\alpha -\beta$ [23], where

$$\begin{array}{l} \alpha =\frac{{\left(S-S^{*}\right)}^{2}}{S^{*}}({I_{m}}^{*}\lambda_{1}+{I_{h}}^{*}\lambda_{2})+\psi H(\frac{I_{h}-{I_{h}}^{*}}{I_{h}})+(\frac{T-T^{*}}{T})(\tau_{1}I_{h}+\tau_{2}I_{m})\\ \;+(\frac{H-H^{*}}{H})(\hbar_{1}I_{m}+\hbar_{2}T), \end{array}$$ (51)

$$\begin{array}{l} \beta =\frac{{\left(S-S^{*}\right)}^{2}}{S^{*}}(r-d_{0})+\psi H^{*}(\frac{I_{h}-{I_{h}}^{*}}{{I_{h}}^{*}})+(\frac{T-T^{*}}{T^{*}})(\tau_{1}{I_{h}}^{*}+\tau_{2}{I_{m}}^{*})\\ \;+(\frac{H-H^{*}}{H^{*}})(\hbar_{1}{I_{m}}^{*}+\hbar_{2}T^{*}), \end{array}$$ (52)

As a result, the having *α*<*β* implies that $\frac{dL_{f}}{dt}<0$ exists simultaneously.


$$\alpha -\beta =0\Rightarrow \frac{dL_{f}}{dt}=0.$$

If *S* = S*, *I*_*h*_ = *I*_h_*, *H* = H*, *T* = T*. Therefore, E* is the greatest compact invariant set for system (2). The Lyapunov LaSalle stability theorem follows. We conclude that in the positive region $R_{+}^{6}\geq 0$, E* is globally asymptotically stable if *α*<*β* for *R*_0_>1.

## 4. Numerical simulations

**Fig 2:**
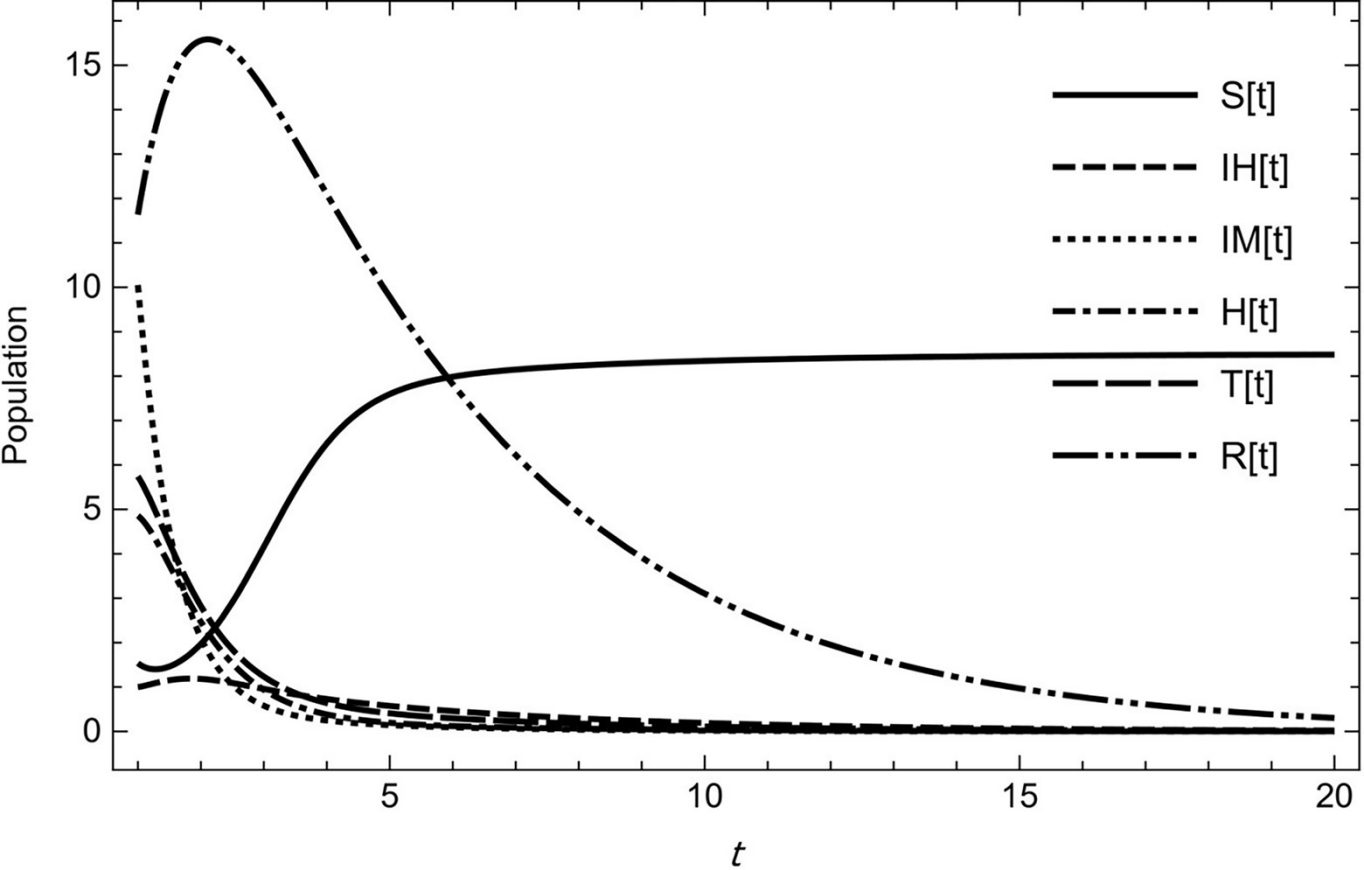
The solution of model (1) asymptotically approaches to the disease free equilibrium for the data (53) with time.

After the mathematical analysis is completed, a numerical simulation is performed to support the theoretical results and demonstrate the model’s behaviour for different initial conditions and combinations of parameter values. This inquiry aims to corroborate our findings and ascertain the impact of changing the parameter values. It has been observed that when considering the following values of the possible set of parameters from Table 1

$$\begin{array}{l} r=2,\;k=10,\;\lambda_{1}=0.2,\;\lambda_{2}=0.12,\;\rho =0.2,\;\psi =.7,\;d_{0}=0.3,\;d_{1}=0.1,\;d_{2}=0.5,\;d_{3}=0.45,\;d_{4}=0.55\\ d_{5}=0.3,\;\tau_{1}=0.75,\;\tau_{2}=0.65,\;\hbar_{1}=0.55,\;\hbar_{2}=0.45,\;r_{1}=0.5,\;r_{2}=0.4,\;r_{3}=0.75,\;r_{4}=0.65. \end{array}$$ (53)

the model (2) approaches asymptotically to the Disease Free Equilibrium and it is simple to indicate that, we have *R*_0_<1 for the data (53) and the solution approaches to *E*^0^ = (8.35,0,0,0,0,0) as shown in Fig 2.

**Table 1:** Details about the *SI*_*h*_*I*_*m*_*THR* model’s symbols and parameter values.

Parameters	Descriptions	Values	References
*r*	Rate at which susceptible individuals grow intrinsically.	2	Fitted
*k*	Carrying capacity of susceptible individuals.	10–30	Fitted
*λ*_1_	Contact rate of HIV Infected with susceptible.	0.2–0.4	[31]
*λ*_2_	Contact rate of Measles Infected with susceptible.	0.1–0.25	[32]
*ρ*	Contact rate of Measles Infected with HIV infected.	0.2–0.4	Fitted
*τ*_1_	Rate at which HIV infected compartment individuals are being treated.	0.75	Fitted
*τ*_2_	Rate at which Measles infected compartment individuals are being treated.	0.65	Fitted
ℏ_1_	Rate at which Measles infected compartment individuals are being hospitalized.	0.5–0.7	Fitted
ℏ_2_	Rate at which Treatment compartment individuals are being hospitalized.	0.4–0.5	Fitted
*ψ*	Rate at which co-infected individuals recover from the Measles disease but still infected with HIV.	0.7	Fitted
*d*_0_	Rate of natural mortality.	0.3	Fitted
*d*_1_	Mortality rate of HIV infected compartment.	0.1–0.4	Fitted
*d*_2_	Mortality rate of Measles infected compartment.	0.5–0.7	Fitted
*d*_3_	Mortality rate of Treatment compartment.	0.45	Fitted
*d*_4_	Mortality rate of Hospitalized compartment.	0.55	Fitted
*d*_5_	Mortality rate of Recovered compartment.	0.3	Fitted
*r*_1_	Recovery rate of HIV infected compartment.	0.5–0.7	Fitted
*r*_2_	Recovery rate of Measles infected compartment.	0.4–0.6	Fitted
*r*_3_	Recovery rate of Treatment compartment.	0.75–0.85	Fitted
*r*_4_	Recovery rate of Hospitalized compartment	0.65	Fitted

On the other hand, the solution of the model (2) approaches the endemic equilibrium point E* = (8.5,0.7,3.3,1.4,1.3,13.35) asymptotically, when considering the following values of the biologically possible set of parameters from Table 1


$$\begin{array}{l} r=2,\;k=20,\;\lambda_{1}=0.32,\;\lambda_{2}=0.2,\;\rho =0.35,\;\psi =.7,\;d_{0}=0.3,\;d_{1}=0.35,\;d_{2}=0.65,\;d_{3}=0.45,\;d_{4}=0.55\\ d_{5}=0.3,\;\tau_{1}=0.75,\;\tau_{2}=0.65,\;\hbar_{1}=0.65,\;\hbar_{2}=0.45,\;r_{1}=0.65,\;r_{2}=0.6,\;r_{3}=0.85,\;r_{4}=0.65. \end{array}$$ (54)

**Fig 3:**
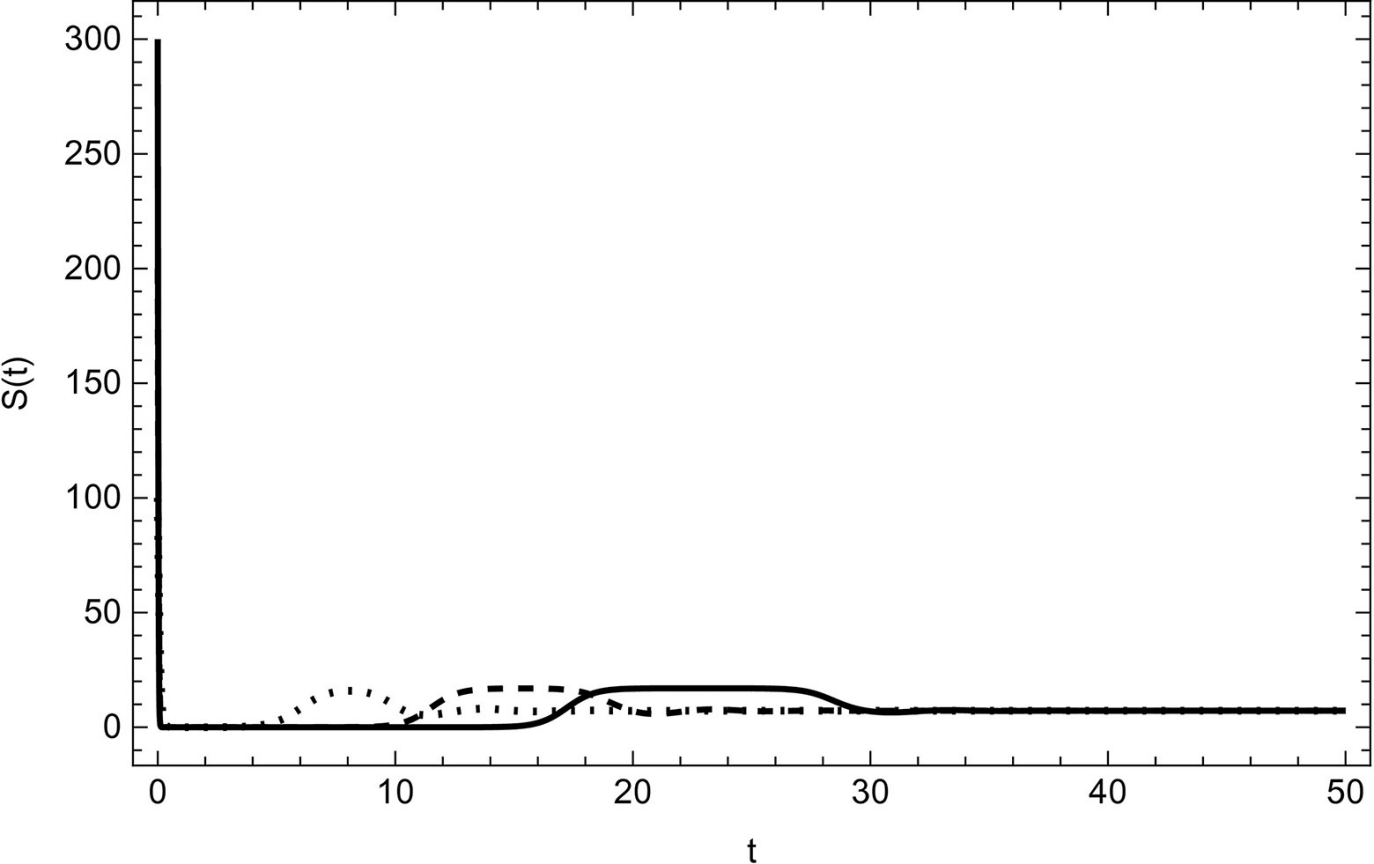
Susceptible compartment asymptotically approaches to the same endemic equilibrium with different initial conditions (100,5,5,0,0,0), (200,10,10,0,0,0), (300,15,15,0,0,0) for the data (54) with time.

**Fig 4:**
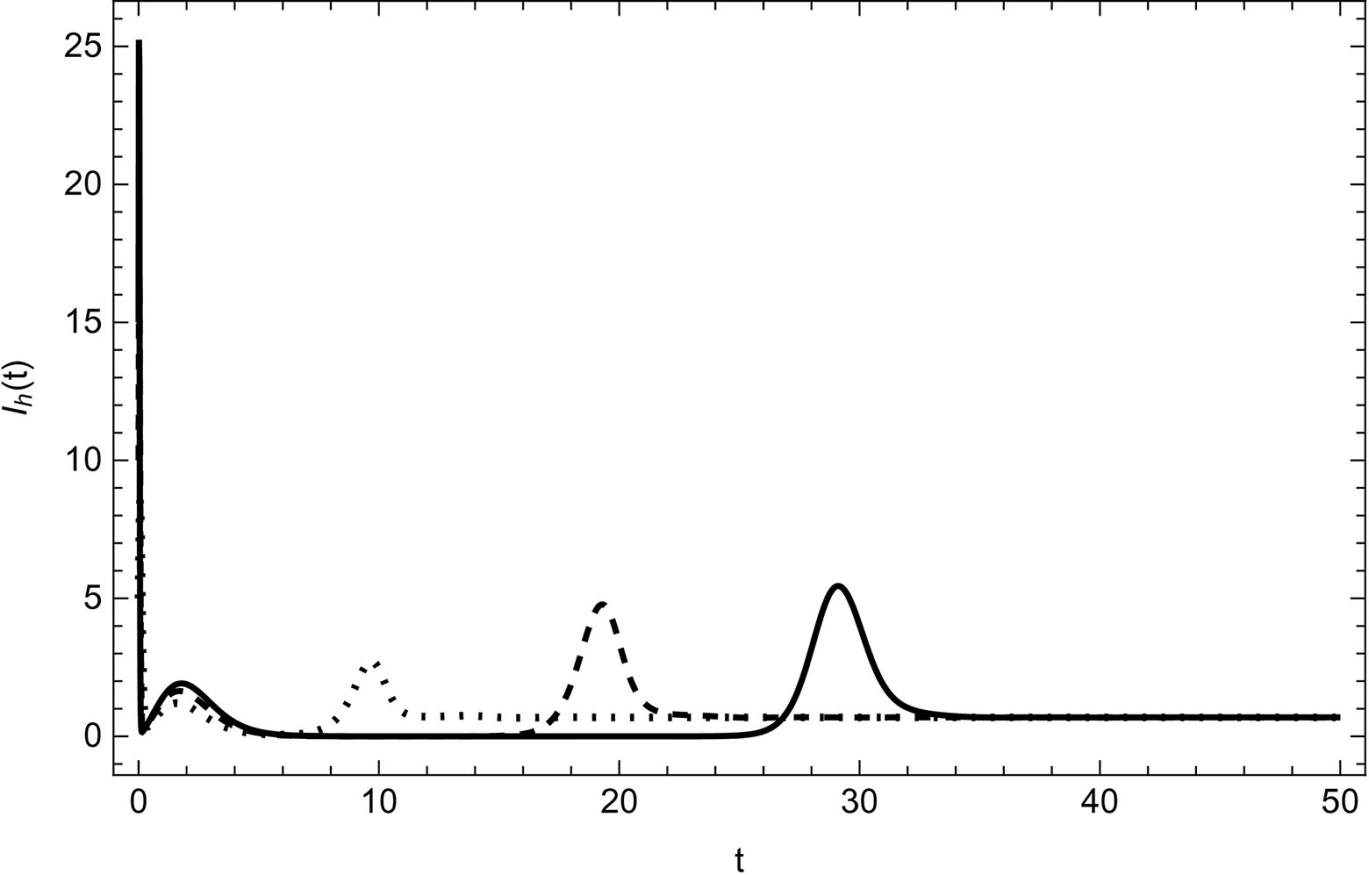
HIV infected compartment asymptotically approaches to the same endemic equilibrium with different initial conditions (100,5,5,0,0,0), (200,10,10,0,0,0), (300,15,15,0,0,0) for the data (54) with time.

**Fig 5:**
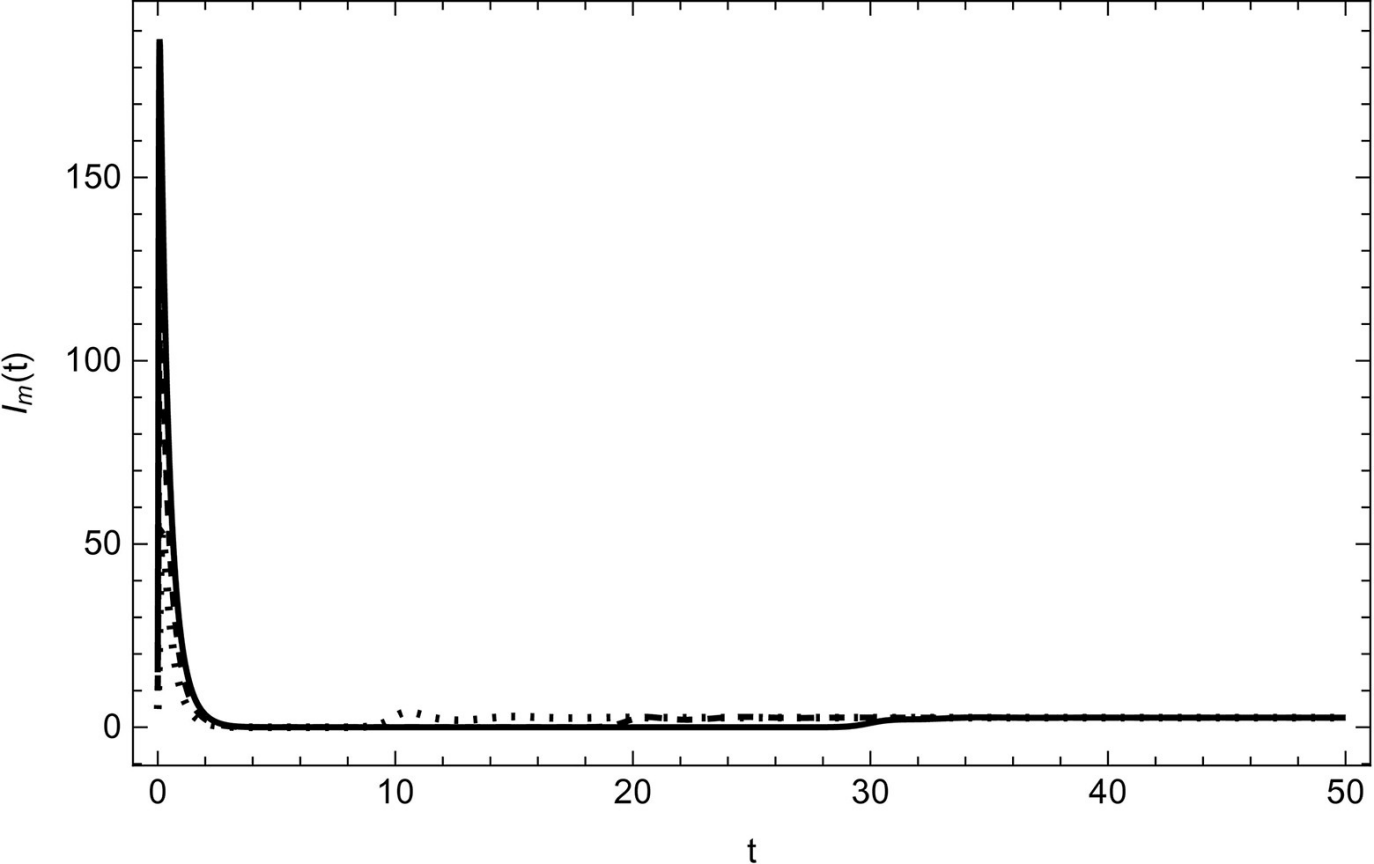
Measles infected compartment asymptotically approaches to the same endemic equilibrium with different initial conditions (100,5,5,0,0,0), (200,10,10,0,0,0), (300,15,15,0,0,0) for the data (54) with time.

**Fig 6:**
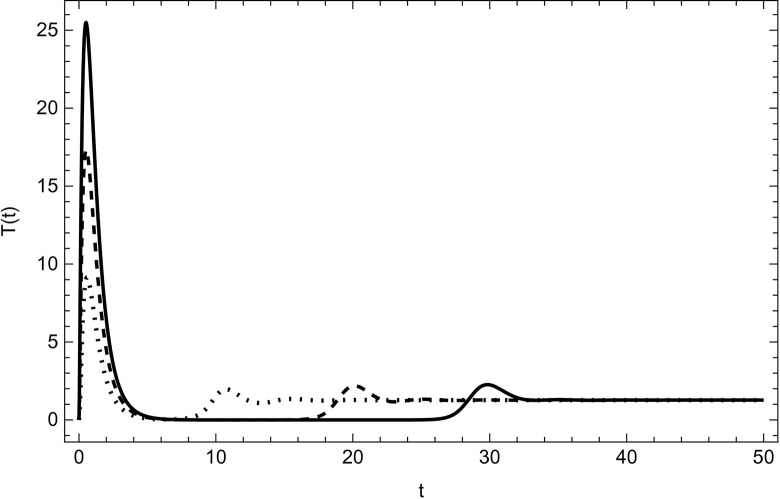
Treatment compartment asymptotically approaches to the same endemic equilibrium with different initial conditions (100,5,5,0,0,0), (200,10,10,0,0,0), (300,15,15,0,0,0) for the data (54) with time.

**Fig 7:**
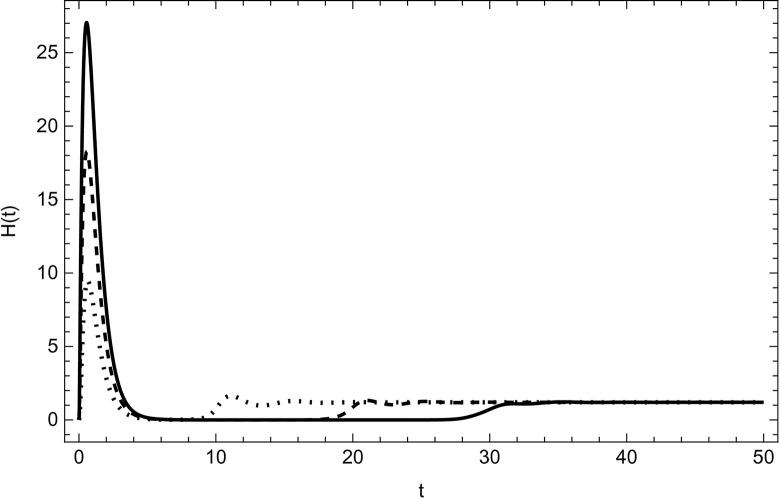
Hospitalized compartment asymptotically approaches to the same endemic equilibrium with different initial conditions (100,5,5,0,0,0), (200,10,10,0,0,0), (300,15,15,0,0,0) for the data (54) with time.

**Fig 8:**
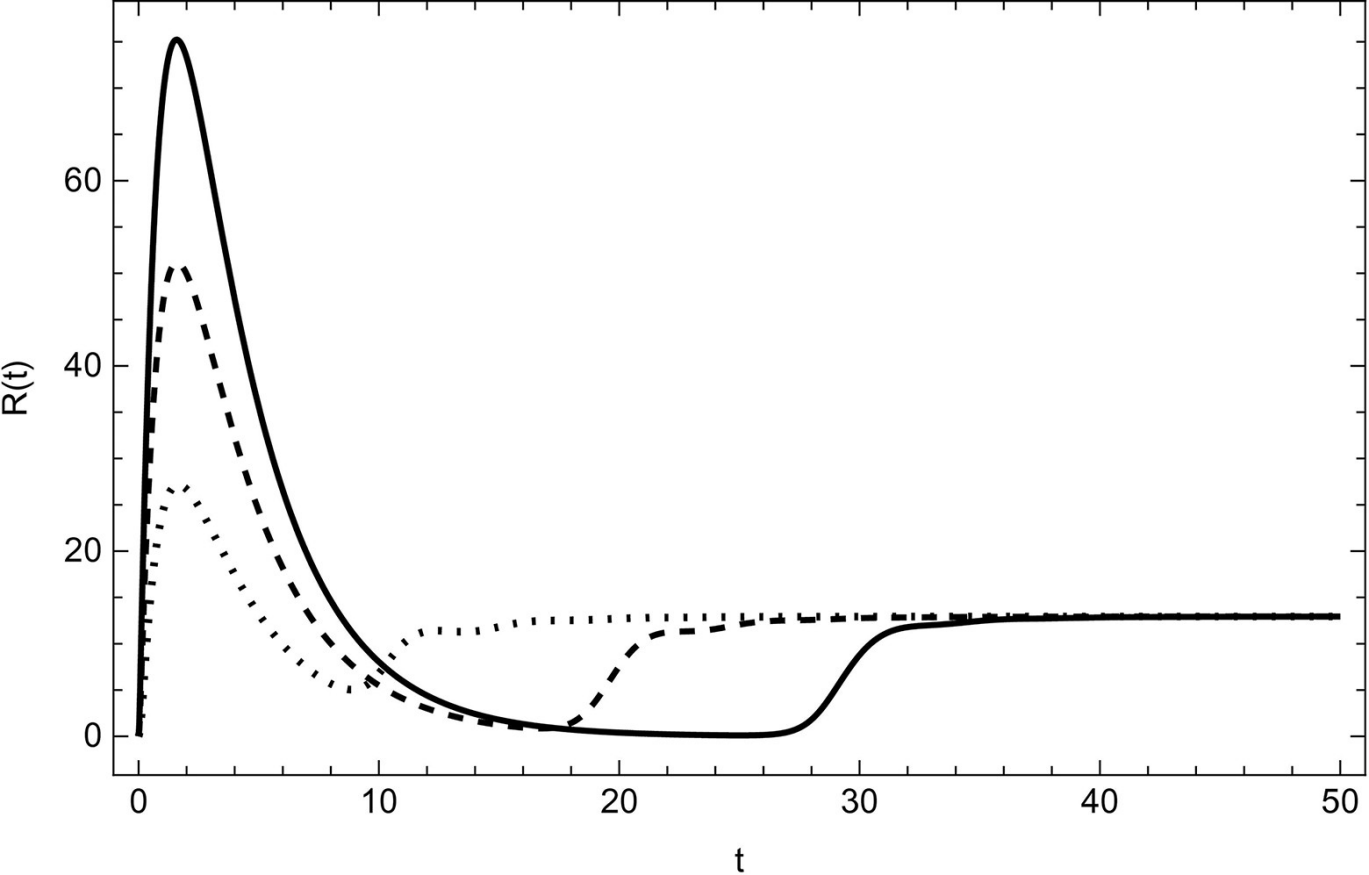
Recovered compartment asymptotically approaches to the same endemic equilibrium with different initial conditions (100,5,5,0,0,0), (200,10,10,0,0,0), (300,15,15,0,0,0) for the data (54) with time.

Started from various starting positions as shown in (Figs 3–8), which amply supports our analytical findings for the data (54) that a globally asymptotically stable positive equilibrium point exists whenever the parameter values are fulfilling the condition *R*_0_>1.

In order to investigate how parameters affect *R*_0_, it is clearly observed that the parameters (*k*,*λ*_1_,*λ*_2_) have positive indices with the reproduction number. This demonstrates the importance of factors such as contact rates, chances of transmission, and infectious carrying capacity in determining how the virus reproduces within a community. An increased risk of disease transmission is shown to directly correlate with increases in parameters (*k*,*λ*_1_,*λ*_2_). So, when their values rise, the basic reproduction number also rises, and infected cases will increase in the population while other parameters remain the same.

Conversely, the parameters $\left(\tau_{1}+\tau_{2}+r_{2}+r_{1}+d_{1}+d_{2}+\hbar_{1}\right)$ show an inverse connection with the reproduction number. This demonstrates that, as the values of these parameters increase while the other parameters remain constant, the value of the reproduction number decreases. This indicates that they reduce the disease’s endemicity or eradicate it from the population.

Now, investigate the impact on the dynamical behaviour of the model (2) by changing one parameter value at a time.

**Fig 9:**
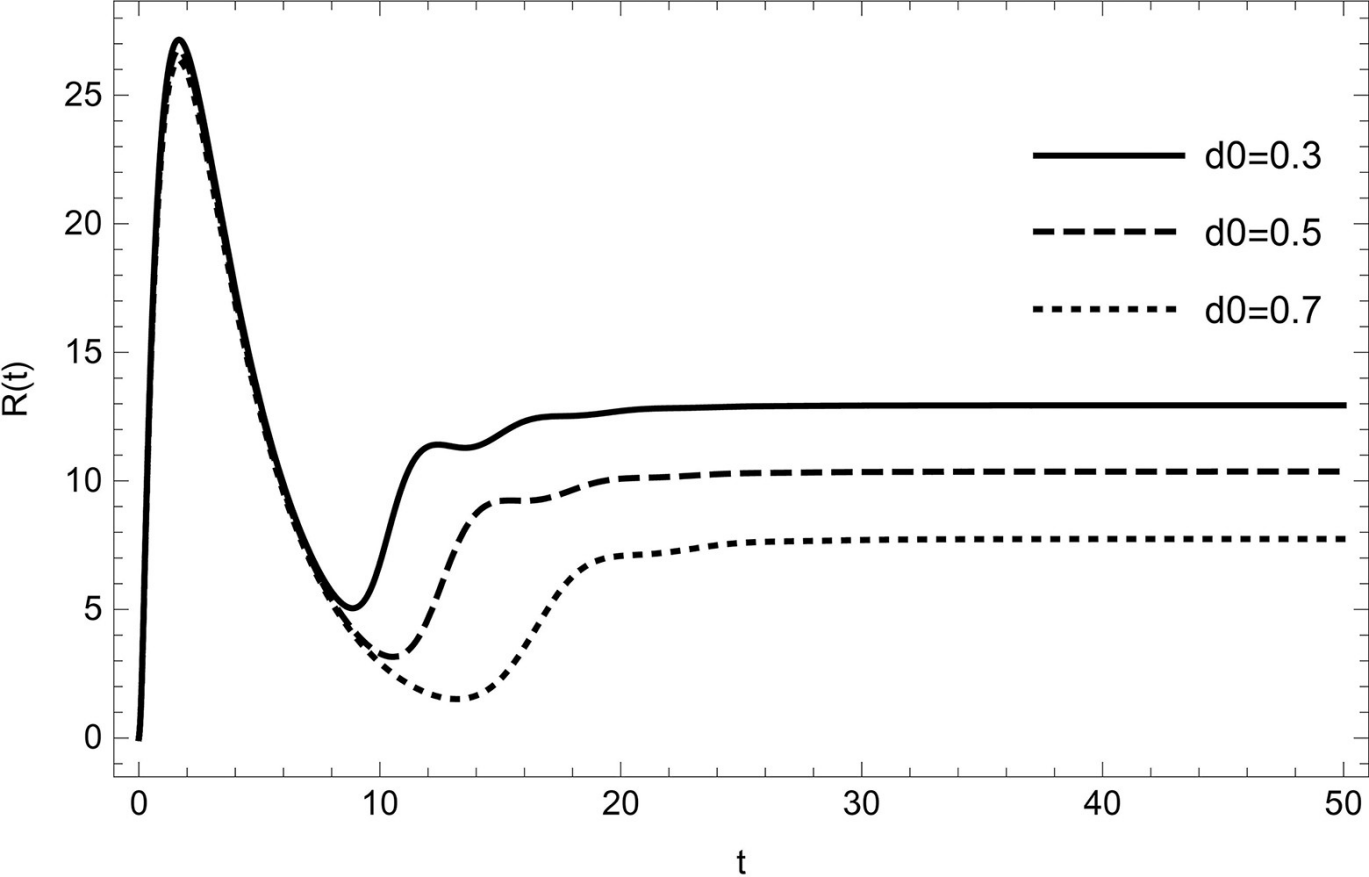
The solution of recovered compartment of model (1) asymptotically approaches to the endemic equilibrium for the data (54), the solution is decreasing with increasing in value of *d*_0_.

**Fig 10:**
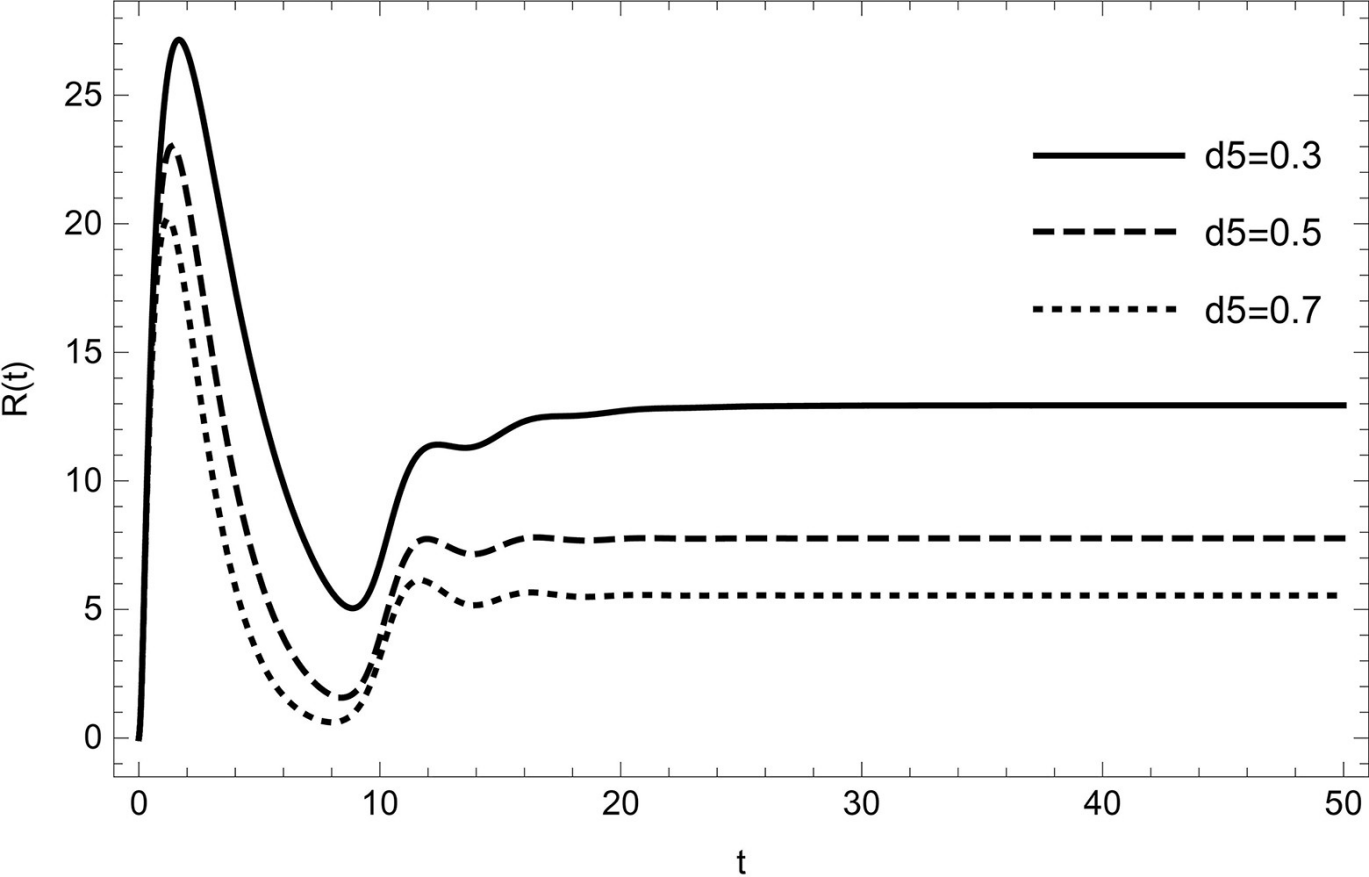
The solution of recovered compartment of model (1) asymptotically approaches to the endemic equilibrium for the data (54), the solution is decreasing with increasing in value of *d*_5_.

**Fig 11:**
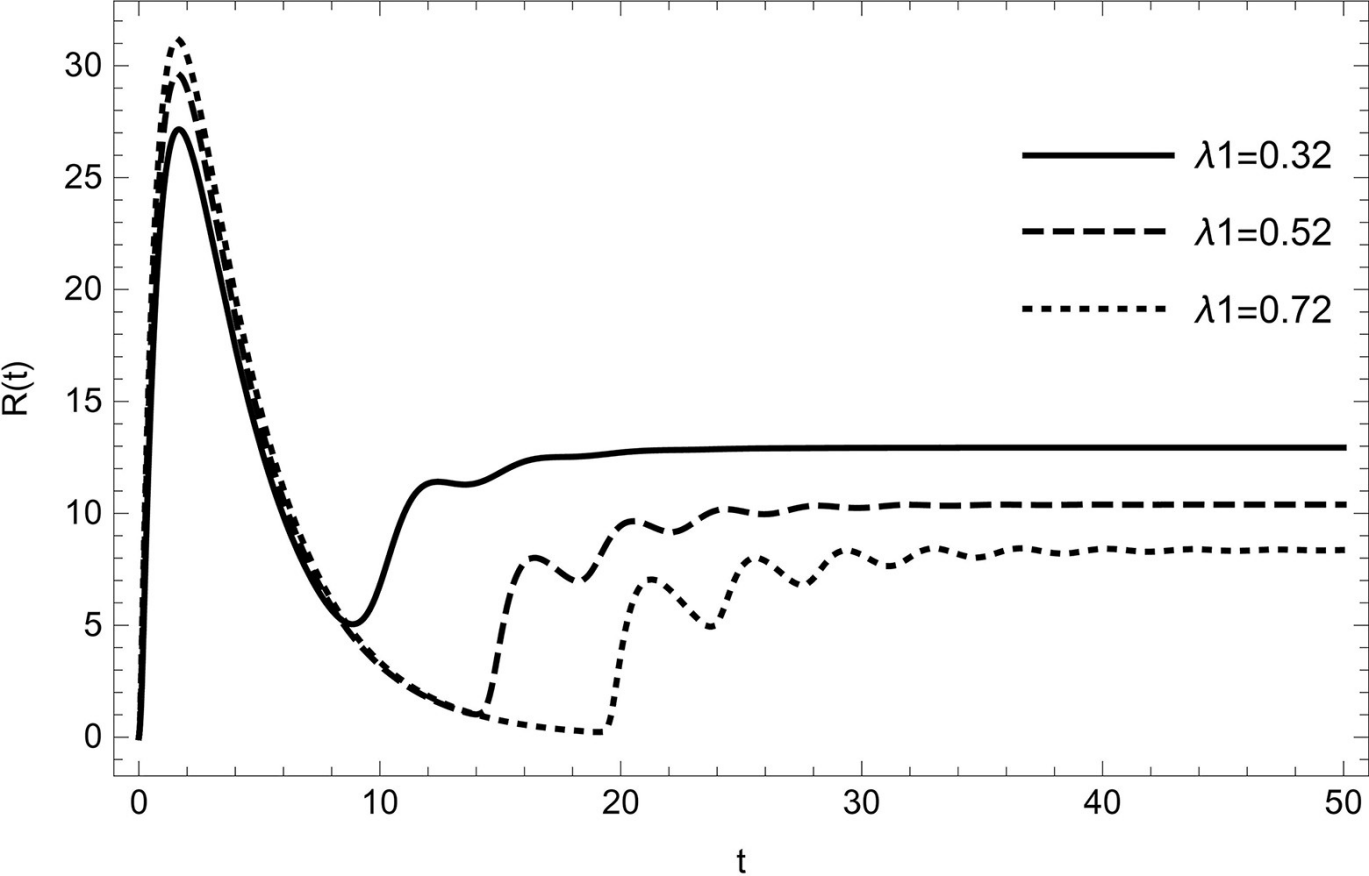
The solution of recovered compartment of model (1) asymptotically approaches to the endemic equilibrium for the data (54), the solution is decreasing with increasing in value of *λ*_1_.

**Fig 12:**
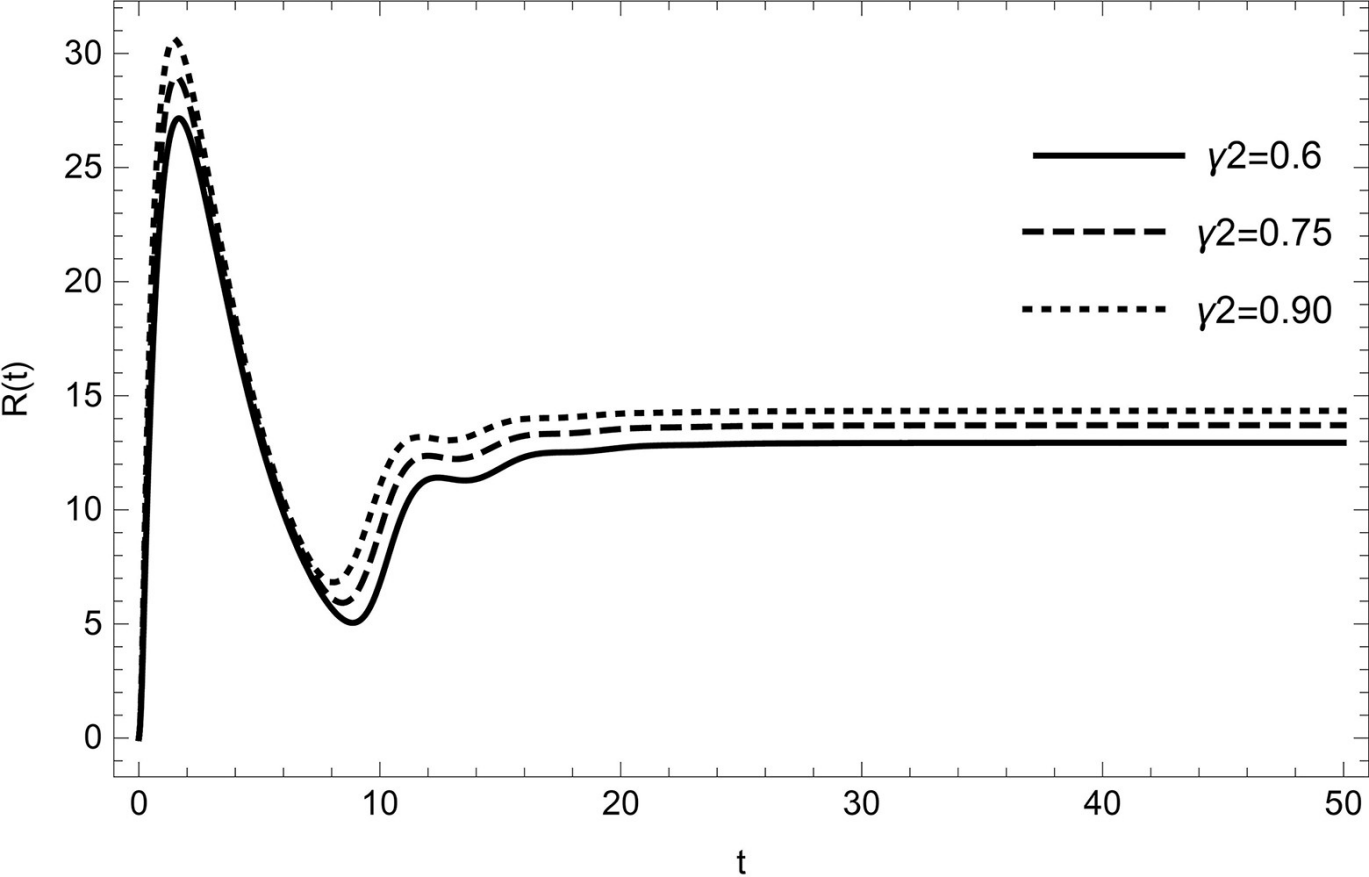
The solution of recovered compartment of model (1) asymptotically approaches to the endemic equilibrium for the data (54), the solution is increasing with increasing in value of *r*_2_.

**Fig 13:**
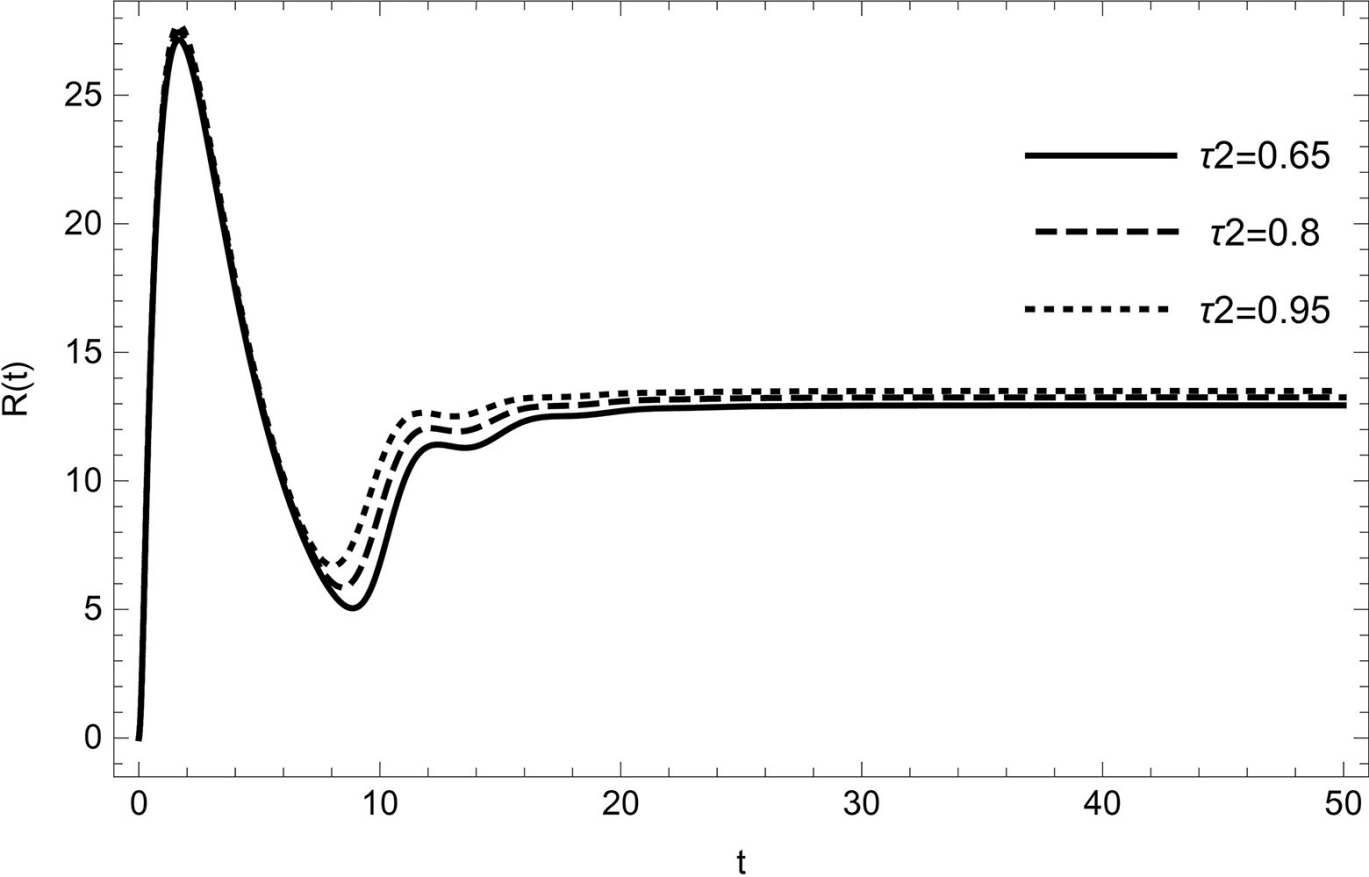
The solution of recovered compartment of model (1) asymptotically approaches to the endemic equilibrium for the data (54), the solution is increasing with increasing in value of *τ*_2_.

**Fig 14:**
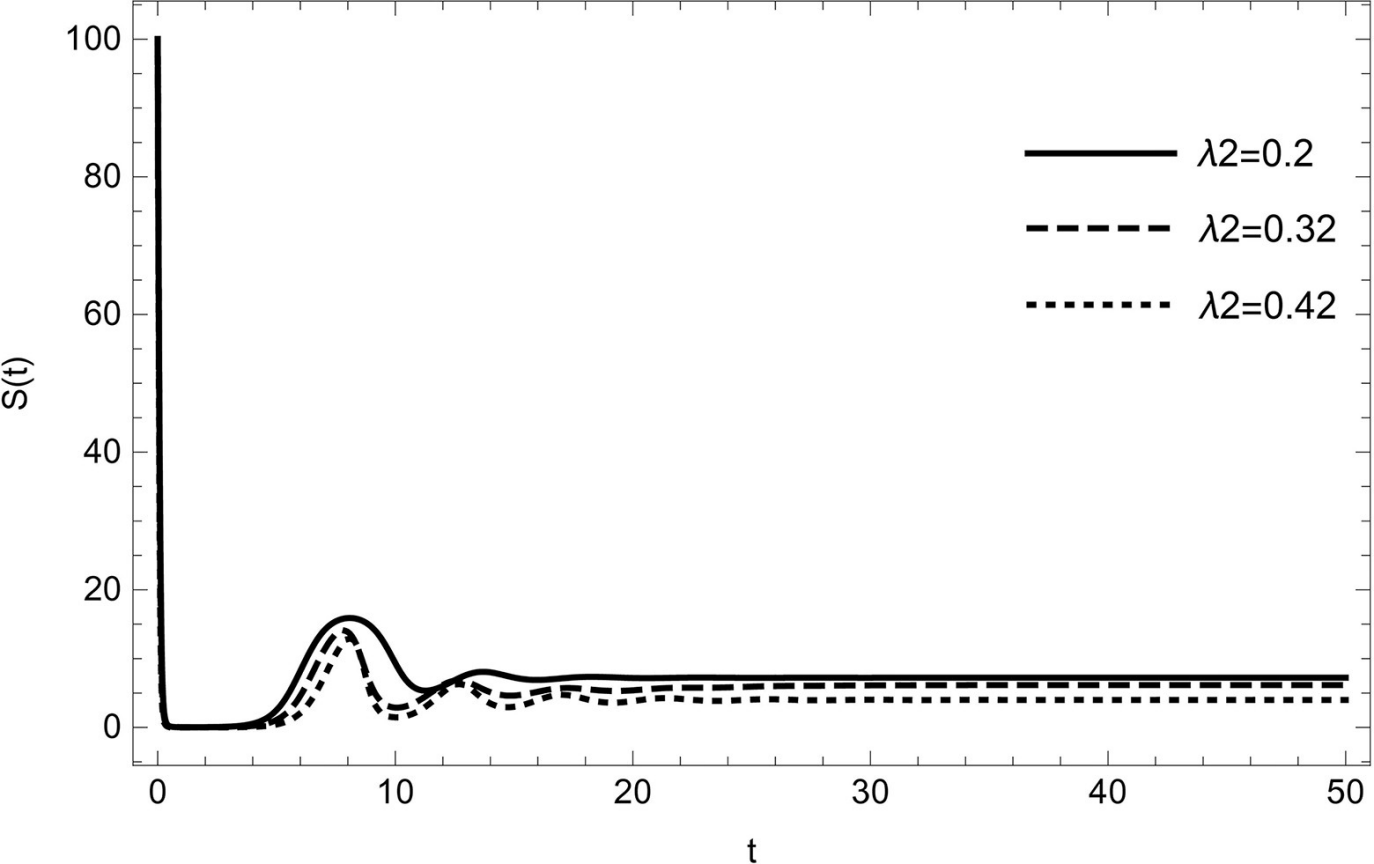
The susceptible compartment changes the dynamics with increasing the number of infected cases of HIV; susceptible are reducing with increasing the value of *λ*_2_.

**Fig 15:**
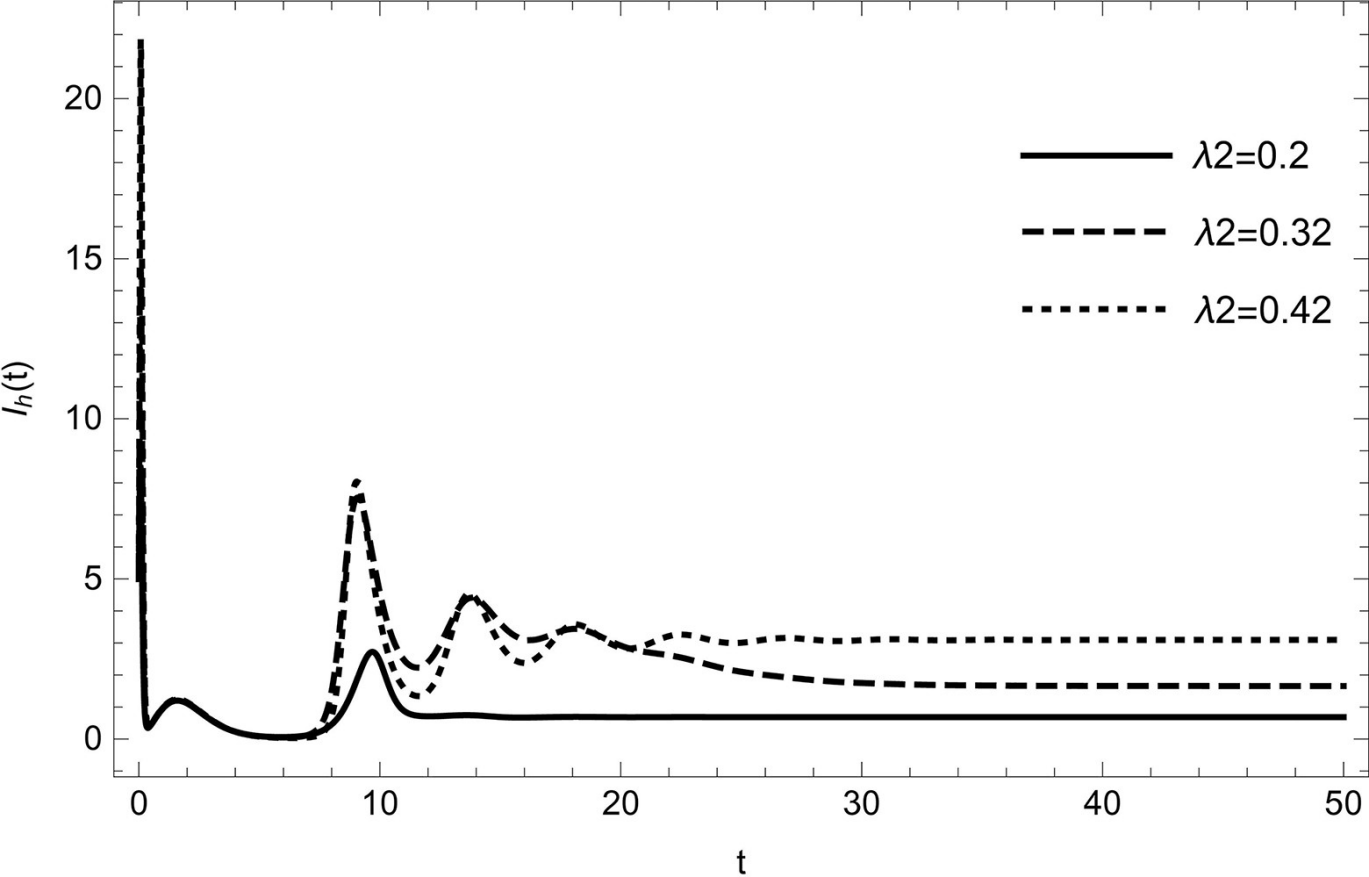
HIV infected compartment changes the dynamics with increasing the number of infected cases of HIV; HIV infected are increasing with increasing the value of *λ*_2_.

**Fig 16:**
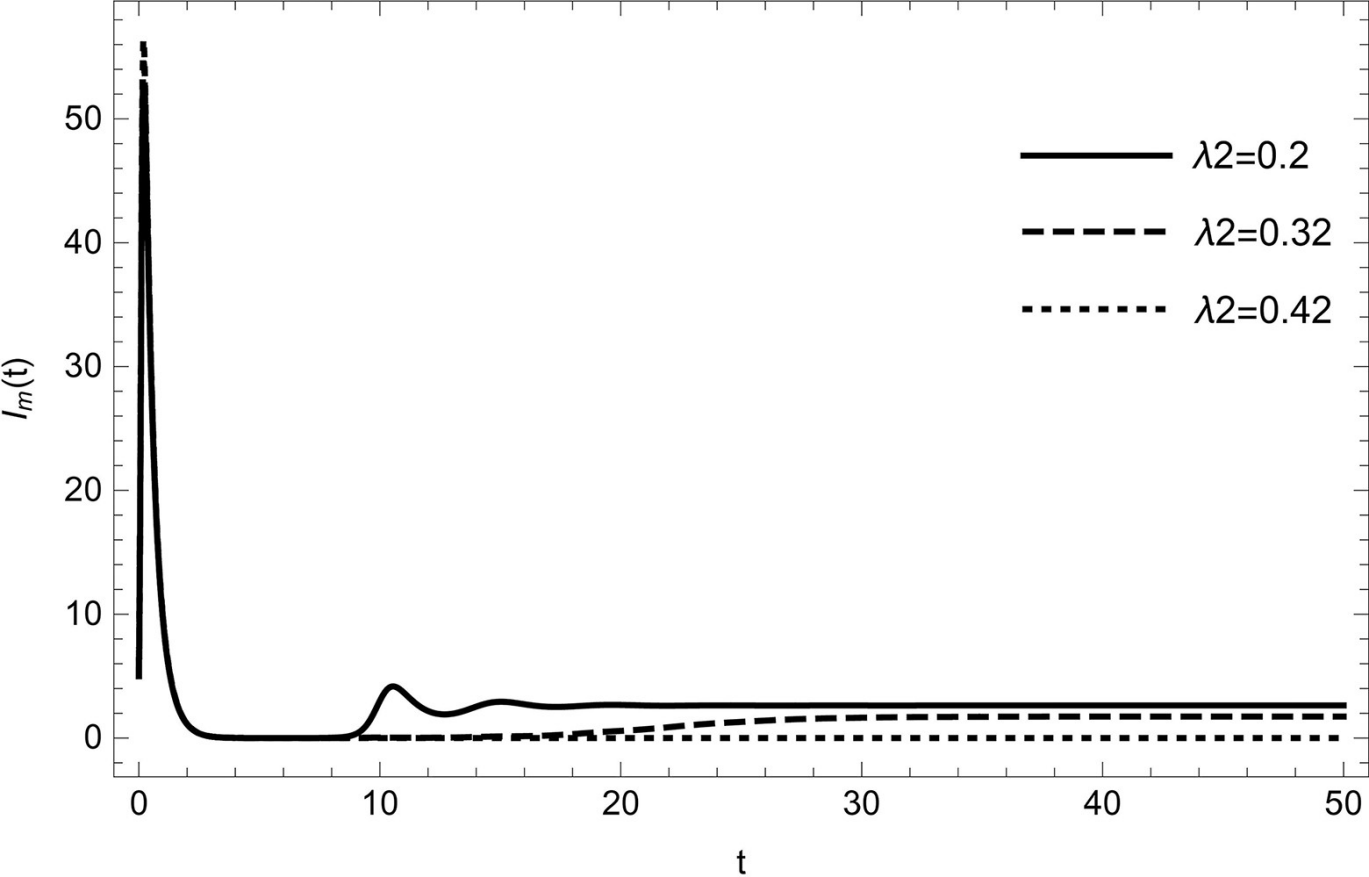
Measles infected compartment changes the dynamics with increasing the number of infected cases of HIV; Measles infected are reducing with increasing the value of *λ*_2_.

**Fig 17:**
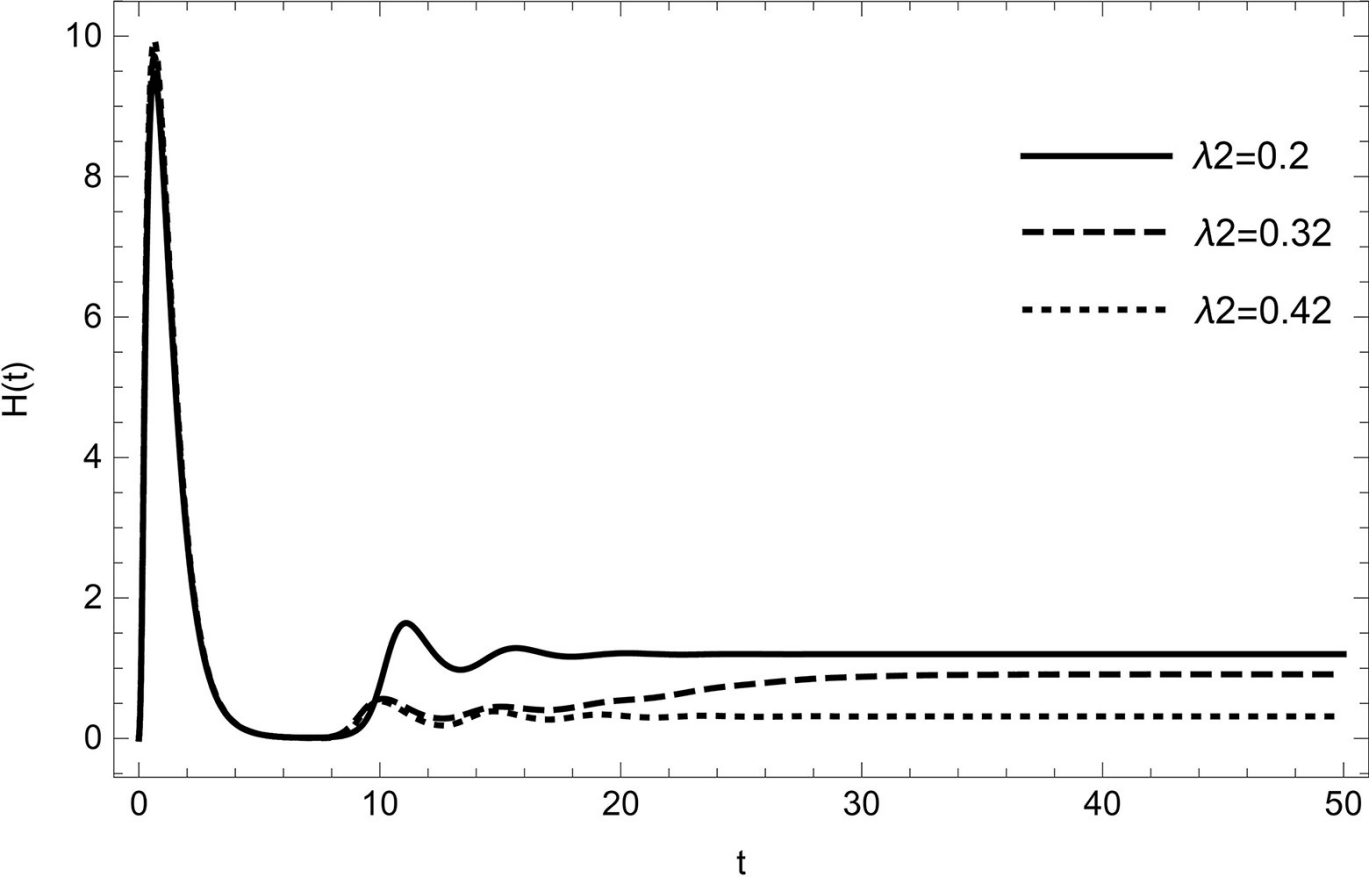
Hospitalized compartment changes the dynamics with increasing the number of infected cases of HIV; Hospitalized population are reducing with increasing the value of *λ*_2_.

**Fig 18:**
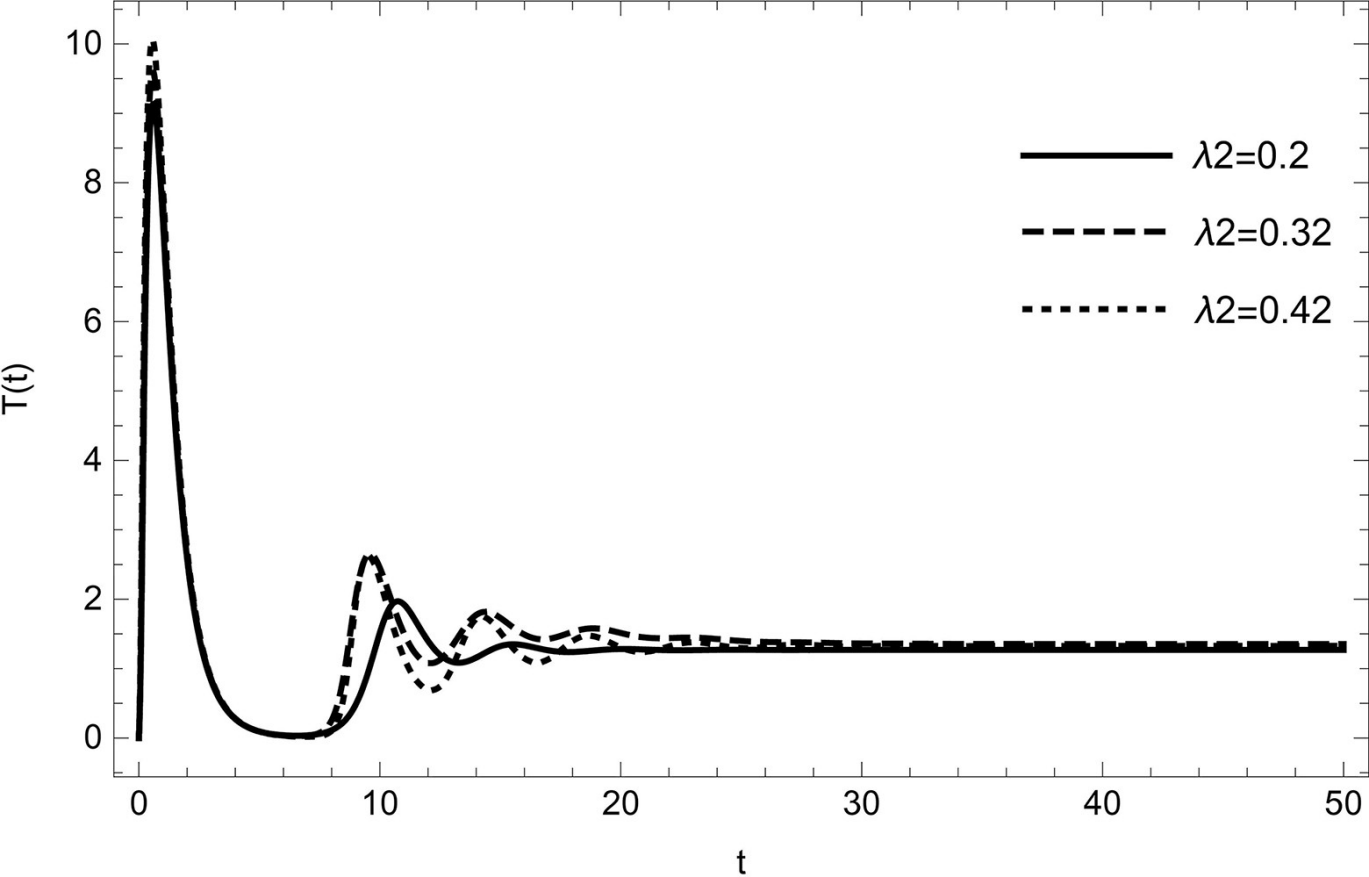
Treatment compartment changes the dynamics with increasing the number of infected cases of HIV; Treatment population are increasing with increasing the value of *λ*_2_.

**Fig 19:**
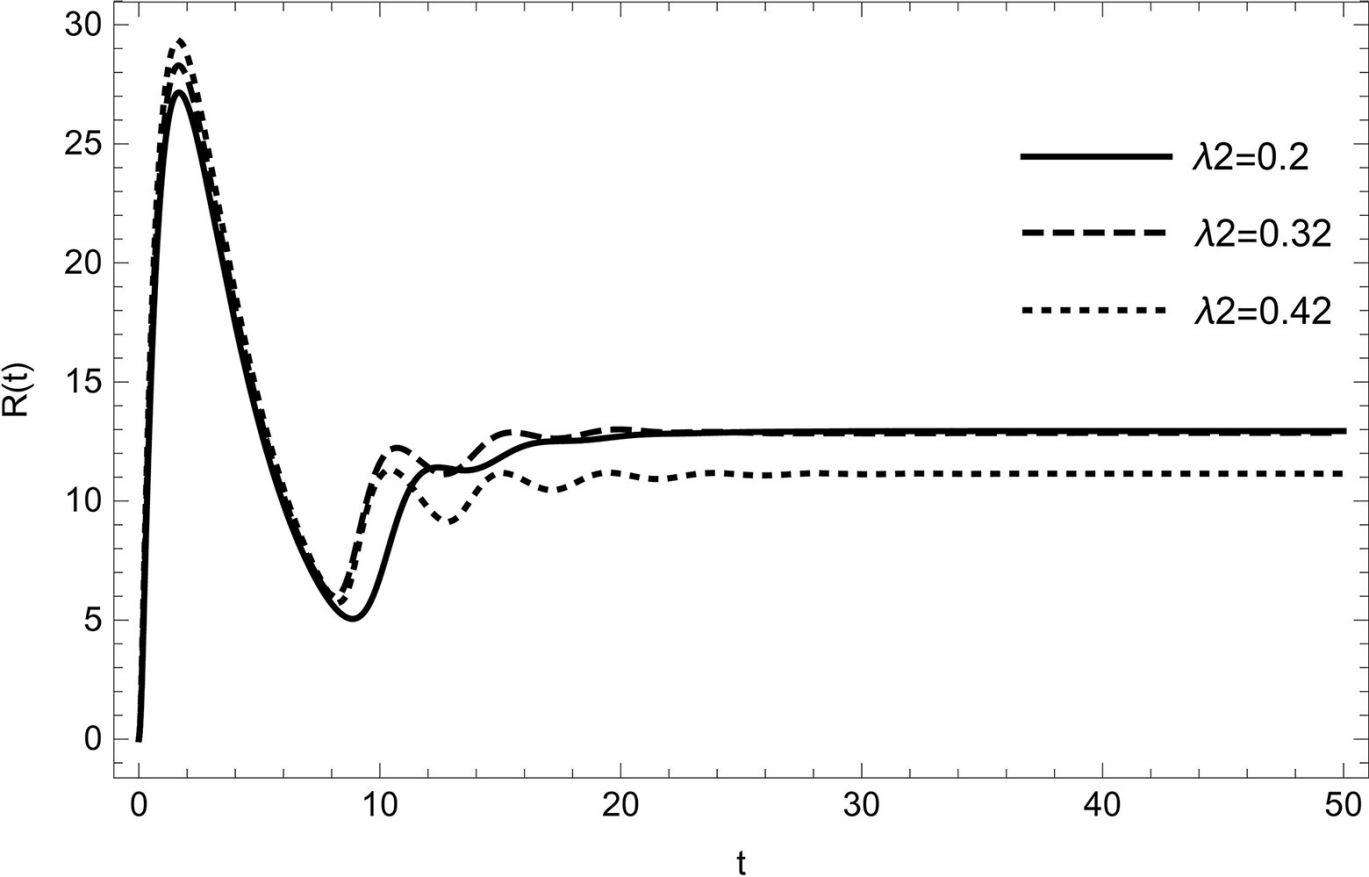
Recovered compartment changes the dynamics with increasing the number of infected cases of HIV; Recovered population are reducing with increasing the value of *λ*_2_.

- The dynamical behaviour is unaffected by changing the parameter values $\left(\tau_{1},\rho ,d_{i}(i=1,2,3,4,5),r_{1},r_{3},r_{4},\hbar_{1},\hbar_{2},\psi \right)$ the system continues to move towards the same equilibrium point.
- Changing the values of the parameters (*λ*_1_,*d*_0_,*d*_5_) affects the dynamical behaviour of the recovery compartment of the model (2). As the parameters start increasing individually, preserving the other parameters fixed in the data (54) gives comparable dynamical behaviour, the recovery cases will start decreasing, as shown in (Figs 9–11).

• Similarly, changing parameters (*τ*_2_,*r*_2_) while maintaining the values of the other parameters unchanged in the data (54), dynamical behaviour affected. As the parameters start increasing individually, the recovery cases will begin increasing along with them, as shown in (Figs 12 and 13) and the endemic population will start reducing.

• The result obtained by varying the contact rate *λ*_2_ of HIV-infected and susceptible while maintaining other parameters unchanged for the data (54) in (Figs 14–19) demonstrates that an increase in the value of the contact rate raises the number of infected people and also decreases the number of cases in hospitalized and recovered compartments. Then, we conclude that the virus dissemination in the population becomes harsher when the contact rate *λ*_2_ is high and the risk of co-infection cases will rise in the population. Therefore, to stop the disease from spreading, all relevant bodies and policymakers must consider ways to reduce the contact between HIV-infected and susceptible.

## 5. Conclusion

It is worth noting that epidemiological models play an essential role in visualising disease transmission dynamics and providing analysis-based recommendations for improved control measures. HIV and measles are the two distinct infectious diseases that often circulate among the populace, and measles can be intense and frequently fatal for patients with HIV infection [6,7]. In our study, we establish a new deterministic mathematical model with logistic growth to examine the dynamics of HIV and measles co-infection in a host population. The model’s basic characteristics, such as uniqueness and existence, have been examined and the boundedness and positivity of the model are also discussed. We queried the model for equilibrium points and determined the reproduction number *R*_0_ by the using next-generation matrix approach. It has been found that diseases are eliminated from the model if the fundamental reproduction number is smaller than unity. The presence of three equilibrium points we demonstrate in our system are: the trivial equilibrium point, which is inherently unstable as all populations cannot perish at once; the endemic and disease-free equilibrium points, which have been investigated utilising Routh-Hurwitz definitions and the Lyapunov function to demonstrate their stability both locally and globally. The endemic and disease-free equilibria are asymptotically stable under the circumstances of *R*_0_>1 and *R*_0_<1. Finally, the model (2) has been numerically solved in order to validate our acquired analytical results and to comprehend the impact of changing each parameter on the overall dynamics of system (1). Based on t behaviour of the numerical solution graphs for various sets of parameters, we conclude the following:

- The parameters $\left(\tau_{1},\rho ,d_{i}(i=1,2,3,4,5),r_{1},r_{3},r_{4},\hbar_{1},\hbar_{2},\psi \right)$ varying one at a time while keeping other parameter unchanged does not affect the dynamical behaviour of the system.
- Model (2) approaches the endemic equilibrium point $E^{*}=(8.5,0.7,3.3,1.4,1.3,13.35)$ asymptotically for a biologically possible set of parameters (54).
- The parameters (*k*,*λ*_1_,*λ*_2_) have a significant impact on the expansion of the disease in the population when they are raised, and while other parameters remain constant, infected cases will increase in the populace.
- The parameters $\left(\tau_{1}+\tau_{2}+r_{2}+r_{1}+d_{1}+d_{2}+\hbar_{1}\right)$ have a positive effect on the eradication of the disease in the populace when they increase, while the other parameters remain constant and the value of the reproduction number decreases.
- The contact rate *λ*_2_ of HIV disease greatly influences the number of infected cases; as the contact rate rise, so does the number of infected cases, and the risk of co-infection cases will rise in the populace.
- Recovery cases will begin increasing along with raising the parameters, (*τ*_2_,*r*_2_)and the endemicity will start reducing among the populace.
- For the data set (53) the model (2) approaches the disease free equilibrium point *E*^0^ = (8.35,0,0,0,0,0) asymptotically.
- The number of recovered cases is significantly influenced by the contact rate of the measles disease *λ*_1_. As the contact rate increases, the number of recovered cases declines.
